# The impact of stroke, cognitive function and post-stroke cognitive impairment (PSCI) on healthcare utilisation in Ireland: a cross-sectional nationally representative study

**DOI:** 10.1186/s12913-022-07837-2

**Published:** 2022-03-29

**Authors:** Isabelle Jeffares, Daniela Rohde, Frank Doyle, Frances Horgan, Anne Hickey

**Affiliations:** 1 Structured Population and Health-Services Research Education Programme (SPHeRE), Division of Population Health Sciences, Royal College of Surgeons in Ireland, Beaux Lane House, Dublin, Ireland; 2 School of Social Work & Social Policy, Trinity College Dublin, Dublin, Ireland; 3 Department of Health Psychology, Division of Population Health Sciences, Royal College of Surgeons in Ireland, Dublin, Ireland; 4 School of Physiotherapy, Royal College of Surgeons in Ireland, Dublin, Ireland

**Keywords:** Healthcare utilisation, Stroke, Cognitive function, Post-stroke cognitive impairment, Older adults, Ireland

## Abstract

**Background:**

Cognitive impairment after stroke is associated with poorer health outcomes and increased need for long-term care. The aim of this study was to determine the impact of stroke, cognitive function and post-stroke cognitive impairment (PSCI) on healthcare utilisation in older adults in Ireland.

**Methods:**

This cross-sectional study involved secondary data analysis of 8,175 community-dwelling adults (50 + years), from wave 1 of *The Irish Longitudinal Study on Ageing (TILDA).* Participants who had been diagnosed with stroke by a doctor were identified through self-report in wave 1. Cognitive function was measured using the Montreal Cognitive Assessment (MoCA). The main outcome of the study was healthcare utilisation, including General Practitioner (GP) visits, emergency department visits, outpatient clinic visits, number of nights admitted to hospital, and use of rehabilitation services. The data were analysed using multivariate adjusted negative binomial regression and logistic regression. Incidence-rate ratios (IRR), odds ratios (OR) and 95% confidence intervals (CI) are presented.

**Results:**

The adjusted regression analyses were based on 5,859 participants who completed a cognitive assessment. After adjusting for demographic and clinical covariates, stroke was independently associated with an increase in GP visits [IRR (95% CI): 1.27 (1.07, 1.50)], and outpatient service utilisation [IRR: 1.49 (1.05, 2.12)]. Although participants with poor cognitive function also visited the GP more frequently than participants with normal cognitive function [IRR: 1.07 (1.04, 1.09)], utilisation of outpatient services was lower in this population [IRR: 0.92 (0.88, 0.97)]. PSCI was also associated with a significant decrease in outpatient service utilisation [IRR: 0.75 (0.57, 0.99)].

**Conclusions:**

Stroke was associated with higher utilisation of GP and outpatient services. While poor cognitive function was also associated with more frequent GP visits, outpatient service utilisation was lower in participants with poor cognitive function, indicating that cognitive impairment may be a barrier to outpatient care. In Ireland, the lack of appropriate neurological or cognitive rehabilitation services appears to result in significant unaddressed need among individuals with cognitive impairment, regardless of stroke status.

**Supplementary Information:**

The online version contains supplementary material available at 10.1186/s12913-022-07837-2.

## Background

Stroke is the second most common cause of death worldwide and a primary reason for acquired incapacity in adults [1]. The global burden of stroke is substantial and continues to increase with improved stroke survival rates and population ageing [1, 2]. Up to 38% of stroke survivors are affected by cognitive impairment one year post-stroke (post-stroke cognitive impairment (PSCI)) [3]; 10% of stroke survivors develop dementia in the first year after stroke [4] and a quarter progress to dementia within three years of initial stroke [5]. Cognitive impairment leads to increased levels of dependency, particularly when accompanied by physical disability [6], and is associated with higher morbidity and mortality risk [7, 8].

Stroke is common in older age, where pre-existing cardiovascular conditions and other comorbidities are most prevalent [4, 9, 10]. Stroke survivors display high rates of healthcare utilisation across a range of services including primary care, inpatient hospital care, outpatient services, social care and rehabilitation [11–13]. Service utilisation is particularly high in the first year after stroke, and over one-third of stroke survivors are re-hospitalised [13, 14]. Factors that predict greater utilisation of inpatient and outpatient hospital care include stroke severity and functional disability [12, 15]. Sociodemographic factors also play a role in healthcare utilisation after stroke; lower socioeconomic status is associated with increased likelihood of hospitalisation after stroke, often precipitated by higher levels of comorbidity and pre-stroke disability in more deprived populations [16].

PSCI is associated with impaired activities of daily living (ADL), which in turn may result in greater utilisation of healthcare services and reduced capacity for independent living [17]. Individuals with PSCI are more likely to be re-admitted to hospital [18] and a recent systematic review found a significant two-fold increase in long-term care admissions of patients with PSCI and dementia [19]. The exact trajectory of PSCI is unpredictable—some stroke survivors show improvement or stable cognitive function over time, while others experience cognitive decline [9, 20]. Hence, access to cognitive assessment, cognitive rehabilitation and secondary prevention, is of considerable importance to prevent recurrent stroke and further cognitive decline [3–5, 21]. Despite the burden of PSCI on healthcare services, the rehabilitation of cognitive impairment has received limited research attention and there is a lack of robust research demonstrating the effectiveness of rehabilitation for PSCI [22]. Nonetheless, cognitive rehabilitation is recommended by stroke rehabilitation guidelines for patients with PSCI [10, 23, 24]. Rehabilitation interventions typically focus on providing the person with compensatory strategies for managing existing deficits with a view to slowing the rate of further cognitive decline, usually involving a combination of restorative and compensatory approaches, individualised to a patient’s specific rehabilitation needs [6]. The aim of cognitive rehabilitation is to improve everyday cognitive function (attention, concentration, memory) and behavioural functioning (managing medications) impacted by stroke [6, 25]. Though limited, evidence for the potential benefits of cognitive rehabilitation in stroke is emerging [26, 27].

Across Europe however, inadequate resourcing constrains the capacity of healthcare teams to deliver cognitive rehabilitation [28, 29], and many services are unable to provide recommended levels of rehabilitation [24, 30]. In the community setting in Ireland, stroke-specific expertise is not readily available or easily accessible and many stroke survivors experience enduring cognitive and psychological impairments [28, 31]. The high prevalence of PSCI and the relative absence of targeted cognitive rehabilitation highlight an area of significant unmet need for stroke patients. Furthermore, improving post-stroke cognitive function has been identified as a key priority among stroke survivors [32].

While stroke and cognitive impairment are associated with poorer health outcomes and increased need for long-term care [11, 19], it is unclear whether these conditions increase healthcare utilisation, independent of other comorbidities and demographic factors. This study aims to address this gap by adjusting for potential confounders and exploring the impact of stroke, cognitive function, and post-stroke cognitive impairment (PSCI) on health service utilisation.

## Methods

### Study design and participants

**Fig. 1:**
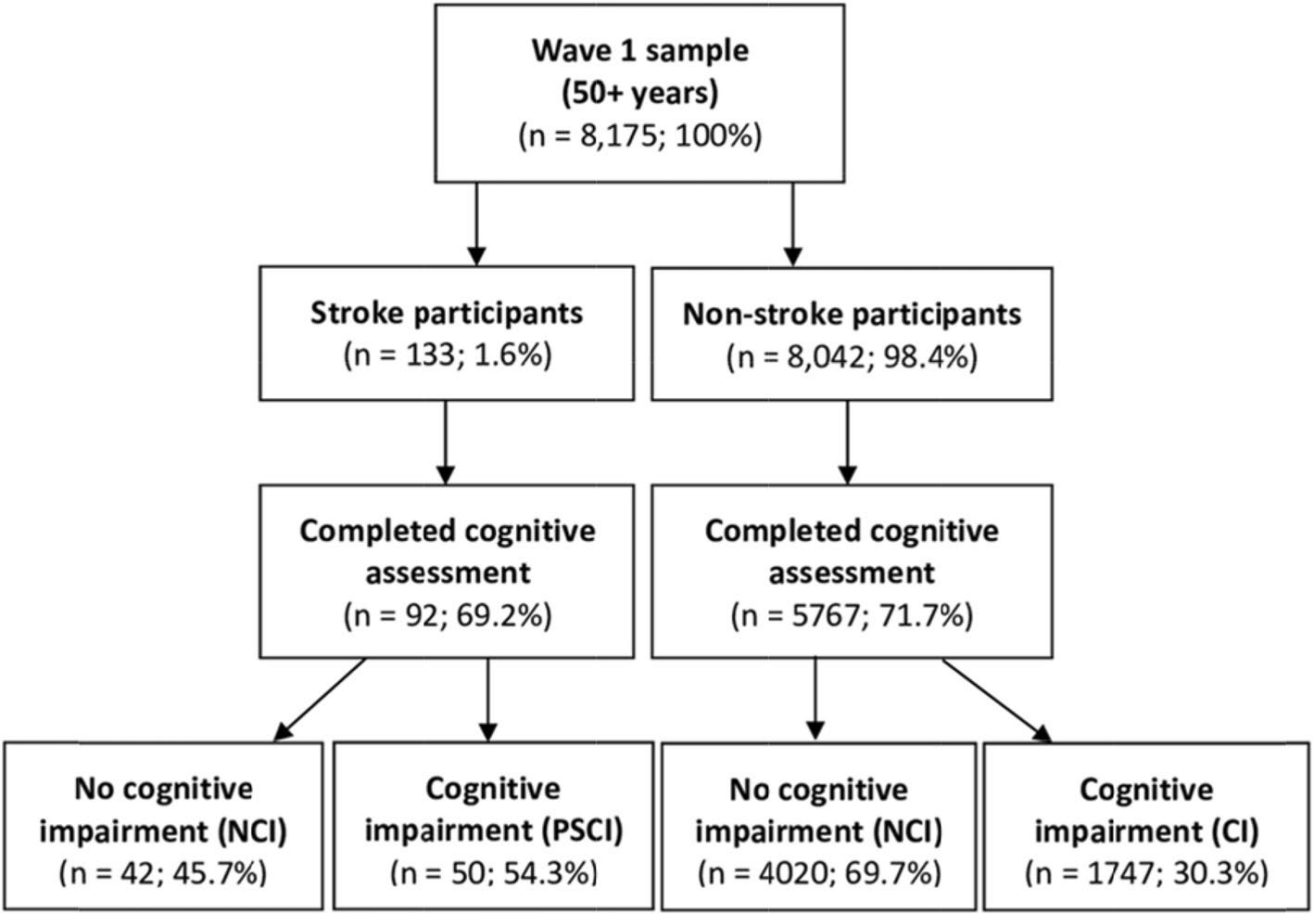
Flow diagram of TILDA participants in Wave 1

*The Irish Longitudinal Study on Ageing (TILDA)* involves a nationally representative sample of 8,175 community-dwelling adults aged 50 years and older in the Republic of Ireland [33]. The present study is a cross-sectional analysis of wave 1 TILDA data, collected between 2009 and 2011 [34]. Cognitive assessment data were available for those who completed a health assessment in wave 1 (*n* = 5,859). Of 133 stroke survivors, 92 completed a cognitive assessment (see Fig. 1). A detailed description of the TILDA methodology is available elsewhere [33, 34]. This study follows the STROBE (Strengthening the Reporting of Observational Studies in Epidemiology) Guidelines [35].

### Exposure variables

#### Stroke status

Participants who had been diagnosed with stroke by a doctor were identified through self-report in wave 1 (*n* = 133). Over one third of participants (*n* = 51, 39%) experienced stroke within the last 3 years and almost 20% (*n* = 25) had experienced more than one stroke in their lifetime. Participants with transient ischaemic attack were not included in the stroke sample.

#### Cognitive function

The Montreal Cognitive Assessment (MoCA) [36] is a global measure of cognitive function, suitable for evaluating cognitive impairment in both stroke and non-stroke populations [36–39]. Cognitive function was measured on a 30-point scale, where lower scores indicate cognitive impairment. A categorical variable was created based on the MoCA cut-off of < 24 to indicate cognitive impairment [40, 41].

### Outcomes

The main outcome of this study was self-reported healthcare use in the previous 12 months, including the number of General Practitioner (GP) visits, emergency department visits, outpatient visits,^1^ and nights admitted to hospital. This study also explored the use of rehabilitation services,^2^ specifically physiotherapy, occupational therapy and psychology.

### Covariates

Covariates included age, sex, education (none/primary school, secondary school/high school, third level/university), employment status (not working or working) and depression (higher scores on the Center for Epidemiological Studies Depression Scale (CES-D)) [42]. Additional covariates included disability status (no disability or (i) disability in activities of daily living (ADL) necessary for basic functioning, e.g., walking, dressing, or (ii) disability in instrumental activities of daily living (IADL) which allow individuals to live independently, e.g., cooking, managing finances or medications). Questions relating to disability were based on validated scales, namely *The Index of Independence in Activities of Daily Living* [43], and *The Lawton Instrumental Activities of Daily Living Scale* [44]. An indicator of socioeconomic status was derived from possession of a medical card/ GP visit card (yes or no). In Ireland, lower income groups often qualify for the medical card, which entitles holders to free or subsidised public healthcare, while the GP visit card permits free GP care for all individuals aged 70 years and older [45].

### Statistical analyses

A combined stroke and cognitive status variable was generated to describe the sample. This variable comprised the following four categories; (i) no stroke, no cognitive impairment, (ii) no stroke, cognitive impairment, (iii) stroke, no cognitive impairment, and (iv) stroke, cognitive impairment. Relationships between the combined exposure variable and categorical covariates (e.g., gender, education, disability status) were explored using chi-square tests, or one-way ANOVA for continuous covariates (e.g., age). As the combined exposure variable included more than two categories, Cramer’s V effect size is reported. Associations between the combined exposure variable and healthcare utilisation (GP visits, emergency visits, outpatient visits and number of nights in hospital) were explored using the Kruskal–Wallis test for non-normally distributed outcomes. Two-tailed tests with an α-level of 0.05 for statistical significance were applied. Chi-square tests were employed to examine utilisation of rehabilitation services, a dichotomous outcome (yes/no).

The continuous MoCA score variable was reverse-scored and converted to a standardized z-score for the regression models, which re-interprets scores in terms of standard deviations from the mean [46]. Multivariate negative binomial regression, reporting incidence-rate ratios (IRR) for effect sizes, was used to model the relationship between stroke, cognitive function and each healthcare utilisation variable separately. This regression technique is suitable for continuous count variables, and is preferred over Poisson regression when data are over-dispersed (variance larger than the mean) [47]. Utilisation of rehabilitation services in the last 12 months (physiotherapy, occupational therapy and psychology) was also explored. These outcomes were combined and modelled as one single variable, which represented use of one or more rehabilitation services. Multivariate logistic regression, reporting odds ratios (OR) for effect sizes, was applied to model use of rehabilitation services. Due to the small numbers utilising rehabilitation services overall, the adjusted *p*-value was reported using Fisher’s exact test.

In the unadjusted and adjusted multivariate analyses, incidence-rate ratios (IRR), odds ratios (OR) and 95% confidence intervals (CI) are presented. Collinearity was investigated by examining the correlation between independent variables; where two variables were highly correlated (e.g., polypharmacy and medical card), the covariate with the lowest correlation with other independent variables in the model, was selected for inclusion in the regression. A number of covariates, namely healthcare coverage, the presence of one or more cardiovascular conditions and the presence of long-term health problems, had negligible effects on the overall results and were excluded from the final regression models (see Additional File 1). The final adjusted models included demographic variables, medical card status, disability (impairment in ADL/ IADL) and depression. The main effects and the interaction between the two exposure variables (stroke and cognitive impairment) were explored in these models. First, healthcare utilisation rates were compared between stroke and non-stroke participants. Second, the impact of varying degrees of cognitive function on healthcare utilisation was evaluated. Finally, the combined impact of stroke status and cognitive function on health service utilisation was explored using interactions. Stata 14 was used to analyse the data [48].

## Results

Table 1 presents the main characteristics of the sample at wave 1. The mean MoCA score at wave 1 was 24.7 (SD 3.78, median 25). Almost one-third (*n* = 1,797; 31%) of the 5,859 participants who completed the MoCA could be classified cognitively impaired, based on a MoCA score of < 24. MoCA scores were available for 92 stroke survivors, more than half of whom had a MoCA score of < 24, indicating PSCI (*n* = 50, 54.4%). Unadjusted comparisons between the reference group (no stroke, no cognitive impairment) and all other groups indicated that stroke survivors (with and without cognitive impairment) were more likely to hold a medical card, be unemployed, take multiple medications, and to have at least one disability (impairment in ADL/IADL). Participants with cognitive impairment (stroke and non-stroke participants) had the lowest MoCA scores, and these participants were more likely to be older, unmarried, living alone and unemployed. Poor cognitive function was also associated with lower levels of education and higher rates of depression. Statistically significant differences between the other stroke/ cognitive groups are presented in Additional File 2.

**Table 1:** Associations between demographic and health variables, and stroke and cognitive status

	**No stroke**		**Stroke**			
	**NCI** **(*n* = 4020; 68.6%)**	**CI** **(*n* = 1747; 29.8%)**	**NCI** **(*n* = 42; 0.7%)**	**CI** **(*n* = 50; 0.9%)**	**Test statistic**	
	**Mean (SD)**	**Mean (SD)**	**Mean (SD)**	**Mean (SD)**	**F**	***P* value**
Montreal Cognitive Assessment Score (MoCA) (n = 5859)	26.7 (1.81)^*^ *n* = 4020 (68.6%)	20.2 (3.10)^*^ *n* = 1747 (29.8%)	25.9 (1.61)^*^ *n* = 42 (0.7%)	18.6 (4.53)^*^ *n* = 50 (0.9%)	3303.95	< 0.001
Age* (*n* = 5849)	61.3 (8.09)^*^ *n* = 4015 (68.7%)	66.3 (9.38)^*^ *n* = 1742 (29.8%)	67.3 (7.05)^*^ *n* = 42 (0.7%)	71.0 (8.93)^*^ *n* = 50 (0.8%)	158.51	< 0.001
	***n* (%)**	***n* (%)**	***n* (%)**	***n* (%)**	**Cramer’s V**	***P* value**
**Sex**						
Male	1832 (45.6)	807 (46.2)	22 (52.4)	28 (56.0)	0.023	0.392
Female	2188 (54.4)	940 (53.8)	20 (47.6)	22 (44.0)		
**Education**						
None/primary school	674 (16.8)^*^	812 (46.5)^*^	12 (28.5)	25 (50.0)^*^	0.244	< 0.001
Secondary/high school	1696 (42.2)	678 (38.9)	17 (40.5)	20 (40.0)		
Third level/university	1650 (41.0)	255 (14.6)	13 (31.0)	5 (10.0)		
**Marital status**						
Not married	973 (24.2)^*^	617 (35.3)^*^	11 (26.2)	22 (44.0)^*^	0.118	< 0.001
Married	3047 (75.8)	1130 (64.7)	31 (73.8)	28 (56.0)		
**Living situation**						
Living alone	700 (17.4)^*^	446 (25.5)^*^	8 (19.0)	17 (34.0)^*^	0.098	< 0.001
Living with others	3320 (82.6)	1301 (74.5)	34 (81.0)	33 (66.0)		
**Geographical location**						
Urban (town/city)	2263 (56.3)^*^	809 (46.4)^*^	27 (64.3)	28 (56.0)	0.093	< 0.001
Rural	1754 (43.7)	936 (53.6)	15 (35.7)	22 (44.0)		
**Employment status**						
Unemployed	2258 (56.2)^*^	1298 (74.3)^*^	35 (83.3)^*^	45 (90.0)^*^	0.182	< 0.001
Employed	1762 (43.8)	449 (25.7)	7 (16.7)	5 (10.0)		
**Medical card**						
No	2602 (64.8)^*^	625 (35.8)^*^	13 (30.9)^*^	4 (8.0)^*^	0.284	< 0.001
Yes	1414 (35.2)	1122 (64.2)	29 (69.1)	46 (92.0)		
**Private insurance**						
No	1207 (30.0)^*^	893 (51.2)^*^	14 (33.3)	32 (64.0)^*^	0.207	< 0.001
Yes	2810 (70.0)	853 (48.8)	28 (66.7)	18 (36.0)		
**Disability status**						
No disability	3.686 (91.7)^*^	1440 (82.4)^*^	29 (69.1)^*^	29 (58.0)^*^	0.168	< 0.001
Disability	334 (8.3)	307 (17.6)	13 (30.9)	21 (42.0)		
**Body Mass Index (BMI)**						
Normal weight	951 (23.7)^*^	366 (21.1)^*^	10 (23.8)	7 (14.6)	0.034	0.081
Overweight/obese	3060 (76.3)	1372 (78.9)	32 (76.2)	41 (85.4)		
**Smoking status**						
Non-smoker/ex-smoker	3426 (85.2)^*^	1419 (81.2)^*^	37 (88.1)	41 (82.0)	0.051	0.002
Current smoker	594 (14.8)	328 (18.8)	5 (11.9)	9 (18.0)		
**Physical activity level**						
Low	1077 (27.0)^*^	630 (36.3)^*^	17 (40.5)	28 (56.0)^*^	0.108	< 0.001
Moderate/High	2908 (73.0)	1103 (63.7)	25 (59.5)	22 (44.0)		
**CVD conditions**						
No CVD conditions	1520 (37.8)^*^	566 (32.4)^*^	2 (4.8)^*^	11 (22.0)^*^	0.080	< 0.001
At least one CVD condition	2500 (62.2)	1181 (67.6)	40 (95.2)	39 (78.0)		
**Medications**						
No Polypharmacy	3493 (87.3)^*^	1262 (73.0)^*^	18 (42.9)^*^	25 (50.0)^*^	0.208	< 0.001
Polypharmacy (5 + medications)	509 (12.7)	466 (27.0)	24 (57.1)	25 (50.0)		
**Depression**						
None/mild	3670 (92.6)^*^	1509 (87.5)^*^	33 (84.6)	40 (83.3)^*^	0.086	< 0.001
Moderate/severe	294 (7.4)	215 (12.5)	6 (15.4)	8 (16.7)		
**Anxiety**						
None/mild	2834 (77.2)^*^	1033 (71.7)^*^	23 (63.9)	22 (64.7)	0.065	< 0.001
Moderate/severe	836 (22.8)	407 (28.3)	13 (36.1)	12 (35.3)		

Results are based on Chi-Square tests (categorical variables) and Analysis of Variance (ANOVA) tests (continuous variables)

*NCI* No Cognitive Impairment, *CI* Cognitive Impairment, *SD* Standard Deviation, *CVD* Cardiovascular Disease

^*^Denotes a statistically significant difference (*p* ≤ 0.05) between this category and the reference category (No stroke/ NCI)

Table 2 presents the unadjusted healthcare utilisation figures by stroke and cognitive status. In comparison with the non-stroke population, participants with stroke utilised all types of healthcare more frequently. Participants with PSCI had significantly higher GP visits, nights in hospital and use of rehabilitation services when compared to the non-stroke cohort (with or without cognitive impairment). Stroke survivors without cognitive impairment demonstrated significantly higher use of emergency services and outpatient services compared to non-stroke participants, but not compared to participants with PSCI. Statistically significant differences between the other stroke/ cognitive groups are presented in Additional File 3.

**Table 2:** Associations between healthcare utilisation variables, and stroke and cognitive function

	**No stroke**		**Stroke**			
**Healthcare utilisation variables**	**NCI** **(*n* = 4020; 68.6%)**	**CI** **(*n* = 1747; 29.8%)**	**NCI** **(*n* = 42; 0.7%)**	**CI** **(*n* = 50; 0.9%)**		
	**N used service (%)** **Mean (SD)** **Median (range)**	**N used service (%)** **Mean (SD)** **Median (range)**	**N used service (%)** **Mean (SD)** **Median (range)**	**N used service (%)** **Mean (SD)** **Median (range)**	**Kruskal–Wallis test**	***P*** **value**
GP visits^a^ (*n* = 5851)	3447 (85.7)	1592 (91.1)	39 (92.9)	48 (96.0)	215.382	< 0.001
	3.2 (3.33)^*^	4.7 (4.65)^*^	6.9 (6.77)^*^	7.3 (6.07)^*^		
	2 (0–25)	4 (0–25)	4 (0–25)	4 (0–25)		
Emergency visits^b^ (*n* = 5856)	563 (14.0)	299 (17.1)	12 (28.6)	12 (24.0)	8.184	0.042
	0.20 (0.62)^*^	0.27 (0.74)^*^	0.62 (1.32)^*^	0.42 (0.88)^*^		
	0 (0–6)	0 (0–6)	0 (0–6)	0 (0–4)		
Number of nights in hospital^c^ (*n* = 5857)	449 (11.2)	259 (14.8)	8 (19.0)	15 (30.0)	11.745	0.008
	0.54 (1.90)^*^	0.84 (2.39)^*^	1.52 (3.42)	2.34 (3.94)^*^		
	0 (0–10)	0 (0–10)	0 (0–10)	0 (0–10)		
Outpatient visits^c^ (*n* = 5857)	1692 (42.1)	769 (44.0)	26 (61.9)	23 (46.0)	15.224	0.002
	1.17 (2.09)^*^	1.32 (2.32)^*^	3.10 (3.57)^*^	1.88 (2.90)		
	0 (0–10)	0 (0–10)	2 (0–10)	0 (0–10)		
	**N (%)**	**N (%)**	**N (%)**	**N (%)**	**Cramer’s V**	***P*** **value**
**Physiotherapy (PT)**						
Not used	3834 (95.4)^*^	1636 (93.7)^*^	40 (95.2)	45 (90.0)	0.041	0.015^d^
Used service	186 (4.6)	111 (6.3)	2 (4.8)	5 (10.0)		
**Occupational therapy (OT)**						
Not used	3977 (98.9)^*^	1721 (98.5)	39 (92.9)^*^	44 (88.0)^*^	0.098	< 0.001
Used service	43 (1.1)	26 (1.5)	3 (7.1)	6 (12.0)		
**Psychology (PSY)**						
Not used	3980 (99.0)	1730 (99.0)	41 (97.6)	50 (100.0)	0.015	0.574^d^
Used service	40 (1.0)	17 (1.0)	1 (2.4)	0 (0.0)		
**At least 1 rehabilitation service used (OT/ PSY)**						
Not used	3782 (94.1)^*^	1608 (92.0)^*^	38 (90.5)	41 (82.0)^*^	0.057	< 0.001^d^
Used services	238 (5.9)	139 (8.0)	4 (9.5)	9 (18.0)		

Results are based on Chi-Square tests (categorical variables) and the Kruskal–Wallis test for non-normally distributed outcomes (continuous variables)

^*^Denotes a statistically significant difference (*p* ≤ 0.05) between this category and the reference category (No stroke/ NCI)

*NCI* No Cognitive Impairment, *CI* Cognitive Impairment, *SD* Standard Deviation, *GP* General Practitioner

^a^variable truncated at 25 visits in the public TILDA dataset; 5126 participants had at least one visit to the GP

^b^variable truncated at 6 visits in the public TILDA dataset; 886 participants had at least one visit to emergency services

^c^variable truncated at 10 visits in the public TILDA dataset; 731 participants spent at least one night in hospital; 2510 participants had at least one visit to outpatient services

^d^Fisher’s exact text (adjusted for small samples)

Table 3 presents the results of the unadjusted and adjusted regression analyses. The unadjusted analyses indicated that stroke survivors and individuals with poor cognitive function had consistently higher rates of healthcare utilisation across all services. In the adjusted models however, no independent associations were identified between stroke or cognitive function and utilisation of emergency services, number of nights in hospital or use of rehabilitation services.

**Table 3:** Unadjusted and adjusted associations between healthcare utilisation, stroke and cognitive function

Healthcare type	Exposure	Unadjusted model IRR (95% CI)	*P* value	Exposure	Fully adjusted model IRR (95% CI)	*P* value
**GP visits**^**a**^						
	**Stroke** (*n* = 8164)	2.04 (1.75–2.38)	< 0.001	**Stroke**	1.27 (1.07–1.50)	0.005
	**Poor cognitive function** (*n* = 5851)	1.24 (1.21–1.27)	< 0.001	**Poor cognitive function**	1.07 (1.04–1.09)	< 0.001
	Stroke*poor cognitive function (*n* = 5851)	0.83 (0.71–0.95)	0.010	Stroke*poor cognitive function	0.94 (0.82–1.08)	0.378
**Emergency visits**^**b**^						
	**Stroke** (*n* = 8167)	2.93 (1.96–4.39)	< 0.001	**Stroke**	1.56 (0.94–2.61)	0.088
	**Poor cognitive function** (*n* = 5856)	1.18 (1.10–1.27)	< 0.001	**Poor cognitive function**	1.06 (0.97–1.15)	0.207
	Stroke*poor cognitive function (*n* = 5856)	0.76 (0.52–1.12)	0.167	Stroke*poor cognitive function	0.94 (0.62–1.43)	0.776
**Nights in hospital**^**c**^						
	**Stroke** (*n* = 8172)	3.88 (1.83–8.23)	< 0.001	**Stroke**	1.93 (0.77–4.81)	0.158
	**Poor cognitive function** (*n* = 5857)	1.26 (1.12–1.41)	< 0.001	**Poor cognitive function**	1.05 (0.93–1.19)	0.461
	Stroke*poor cognitive function (*n* = 5857)	0.92 (0.44–1.90)	0.814	Stroke*poor cognitive function	1.03 (0.48–2.22)	0.948
**Outpatient visits**^**d**^						
	**Stroke** (*n* = 8168)	2.05 (1.51–2.79)	< 0.001	**Stroke**	1.49 (1.05–2.12)	0.025
	**Poor cognitive function** (*n* = 5857)	1.05 (1.00–1.10)	0.060	**Poor cognitive function**	0.92 (0.88–0.97)	0.003
	Stroke*poor cognitive function (n = 5857)	0.71 (0.54–0.95)	0.019	Stroke*poor cognitive function	0.75 (0.57–0.99)	0.039
**Healthcare type**	**Exposure**	**Unadjusted model** **OR (95% CI)**	**P value**	**Exposure**	**Fully adjusted model** **OR (95% CI)**	**P value**
**Rehabilitation services used**^e^						
	**Stroke** (*n* = 8175)	2.70 (1.68–4.34)	< 0.001	**Stroke**	1.25 (0.64–2.43)	0.514
	**Poor cognitive function** (*n* = 5859)	1.21 (1.10–1.33)	< 0.001	**Poor cognitive function**	0.99 (0.88–1.11)	0.804
	Stroke*poor cognitive function (*n* = 6113)	1.07 (0.71–1.62)	0.734	Stroke*poor cognitive function	1.33 (0.85–2.08)	0.216

Full model adjusted for health and demographic factors (stroke status, cognitive function, age, sex, education, employment, medical card, disability and depression)

Poor cognitive function is based on the continuous Montreal Cognitive Assessment (MoCA) score. Stroke*poor cognitive function tests whether there is an interaction between stroke status and cognitive function

*IRR* Incidence-rate ratio, *OR* Odds Ratio, *GP* General Practitioner

^a^Fully adjusted model (*n* = 5753)

^b^Fully adjusted model (*n* = 5757)

^cd^Fully adjusted model (*n* = 5758)

^e^Fully adjusted model (*n* = 5760)

In the adjusted multivariate regression, GP visits were significantly higher in stroke after adjusting for cognitive function, demographic factors, medical card entitlement, disability and depression [IRR (95% CI): 1.27 (1.07, 1.50)]. A significant independent association was also found for cognitive function and GP utilisation, indicating that a one standard deviation decrease in cognitive score was associated with a 7% increase in GP visits [IRR (95% CI): 1.07 (1.04, 1.09)]. The interaction between stroke and cognition was not significant for GP visits in the adjusted model.

**Fig. 2:**
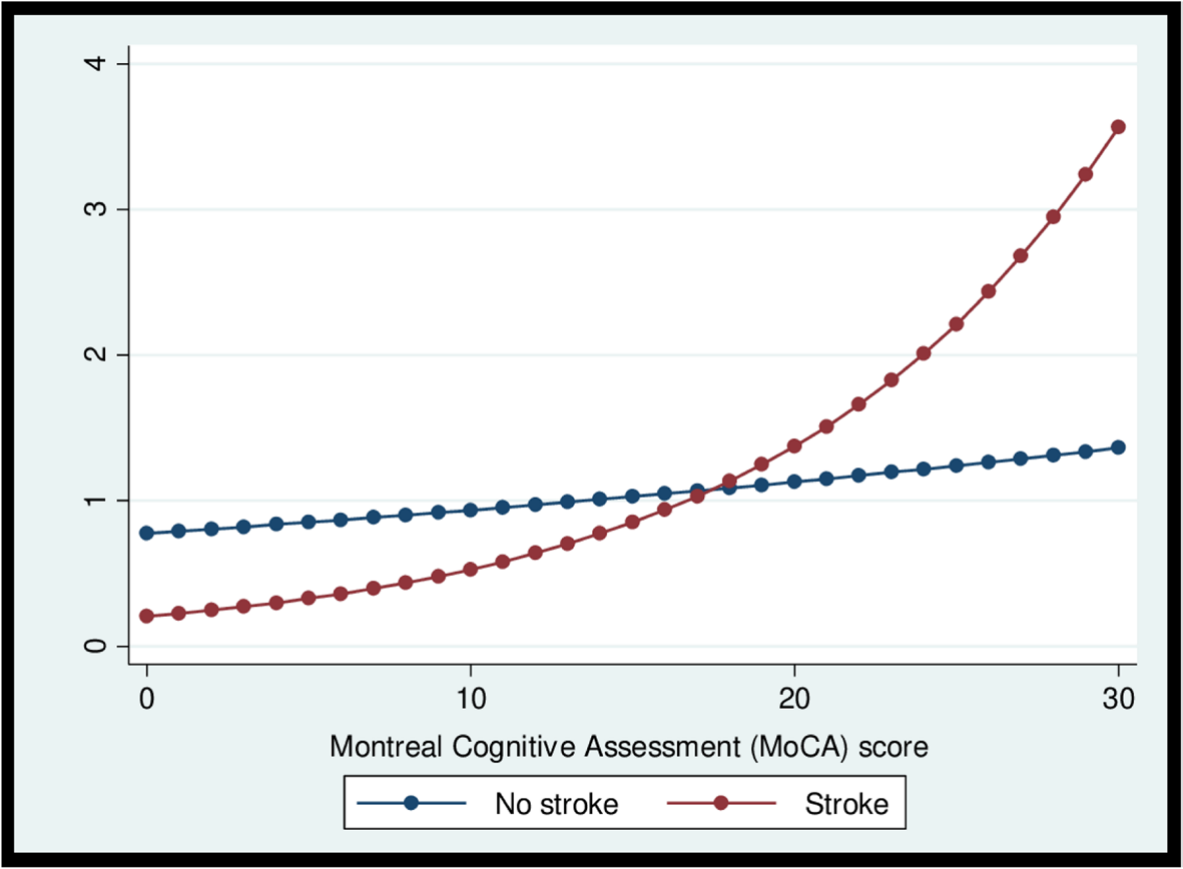
Interaction between stroke and cognitive function on Outpatient services utilisation

Stroke survivors were almost 50% more likely to visit outpatient services, after adjusting for confounders [IRR (95% CI): 1.49 (1.05, 2.12)] regardless of cognitive impairment status. In contrast, respondents with poor cognitive function were less likely to visit outpatient services [adjusted IRR (95% CI): 0.92 (0.88, 0.97)]. The interaction between stroke and cognitive function was significant for outpatient visits in the adjusted model [IRR (95% CI): 0.75 (0.57, 0.99)]. Figure 2 illustrates that higher levels of cognitive function were associated with a sharp increase in outpatient visits among stroke survivors, while a more gradual increase was observed for non-stroke participants. Moreover, higher levels of PSCI (lowest MoCA scores) were associated with the smallest number of outpatient visits.

The analysis was repeated using the dichotomised Montreal Cognitive Assessment (MoCA) variable (scores < 24 indicate cognitive impairment) and these results are presented in Additional File 4. The results were similar, except in the case of outpatient visits, where there was no difference in outpatient service utilisation among those with or without cognitive impairment. In contrast, the results of the original analysis (using the continuous MoCA score) indicated that poor cognitive function was associated with reduced outpatient service utilisation. These results demonstrate the power of using continuous variables in regression analysis, where utilisation estimates are derived from the full range of cognitive scores.

## Discussion

This study aimed to evaluate the impact of stroke, cognitive function, and post-stroke cognitive impairment (PSCI) on health service utilisation. The results of the adjusted regression analysis indicated that stroke was associated with higher utilisation of GP and outpatient services. Participants with poor cognitive function also visited the GP more frequently; however, utilisation of outpatient services was significantly lower in this group. Furthermore, the combined impact of stroke and poor cognitive function (PSCI) was associated with the fewest visits to outpatient services. While stroke and poor cognitive function were associated with increased emergency services utilisation in the unadjusted models, disability and depression appeared to mediate emergency visits in the adjusted analysis. Stroke survivors and individuals with cognitive impairment are commonly affected by disability and depression [15, 19, 49], which may explain why no significant association was found once these confounders were included in the model. The number of nights spent in hospital also failed to reach significance in the adjusted regression analysis. Stroke and cognitive impairment are more common in older age [4, 9] and, in this study, older age appeared to mediate the number of nights spent in hospital by participants with cognitive impairment.

Stroke survivors and participants with poor cognitive function had the highest rates of GP utilisation. Stroke and cognitive impairment are more common in older age and among those with additional comorbidities (e.g., high blood pressure, diabetes and psychological distress), leading to increased healthcare need [4, 9, 10]. Furthermore, a large proportion of the Irish population over the age of 70 have a medical card or GP visit card [45], which entitles them to free or subsidised healthcare. In the present study however, GP visits remained higher even after adjusting for disability, depressive symptoms and medical card entitlement. GPs are often the first point of contact for stroke survivors discharged to the community [11], and these practitioners are responsible for managing a stroke patient’s secondary prevention treatment, identifying post-stroke rehabilitation needs and facilitating referral to appropriate services [10, 23, 50]. However, research suggests that these recommendations are not routinely implemented in practice [51, 52] and patients with complex post-stroke cognitive and psychological difficulties often require stroke-specific expertise [53].

Stroke survivors also visited outpatient clinics more frequently, highlighting the role of these services in post-stroke follow-up [54]. In Ireland, outpatient services provide diagnostic tests such as X-Rays, specialist consultation, stroke-specific treatments such as warfarin clinics, and rehabilitation services, which do not require hospital admission [55]. Research suggests that stroke severity and functional disability predict higher utilisation of outpatient care [15]. In contrast, poor cognitive function and PSCI were associated with a significant decrease in outpatient service use, highlighting that cognitive impairment, with or without stroke, may be a significant barrier to accessing these services. While these results need to be replicated in prospective studies, the findings are consistent with other research [56, 57], which reports that cognitive impairment makes it difficult for an individual to identify his or her own healthcare needs, often due to reduced insight. These patients may be more reliant on caregivers for support in terms of arranging appointments and accessing services. They may also encounter other barriers to outpatient care, including poor availability of services and delays to accessing treatment [57–59], in addition to logistical barriers such as limited transport options and parking fees [60].

The TILDA study reported that older participants (80 + years of age) had significantly fewer outpatient visits compared to younger participants [61]. Frailty is prevalent among individuals with cognitive impairment and dementia [62, 63], particularly in the context of stroke, and this may explain why utilisation was lower among these participants. Less frequent visits to outpatient services may account for increased GP utilisation among participants with poor cognitive function in this study. Outpatient services frequently do not adequately address the needs of cognitively impaired populations and, as a result, these patients are primarily managed by GPs in the community [10, 23]. Considering the high prevalence of PSCI and potential for further cognitive decline and progression to dementia, access to cognitive assessment and regular patient follow-up in the community is of considerable importance [3, 4].

In this study, rehabilitation services were defined as therapies delivered through community care services, which can be accessed via the GP or self-referral through local health centres. These services are freely available for medical cardholders, while user fees apply to individuals not covered by the medical card. After adjusting for confounders, stroke and cognitive function had no significant impact on visits to community-based rehabilitation services. However, it was not possible to identify whether participants received rehabilitation through hospital outpatient services, and this may have affected the numbers accessing community rehabilitation services. Additionally utilisation rates were low overall, which is not necessarily indicative of existing demand for rehabilitation but rather may reflect service availability. In Ireland, access to psychology, neuropsychology and cognitive rehabilitation is extremely limited for people with stroke [54, 64, 65]. Stroke-specific expertise is not easily accessible in the community and many stroke survivors are never referred to these services [64], or are unable to access them due to long waiting lists [58, 59]. Increased investment in the development of specialist community stroke rehabilitation teams could support GPs in the long-term management of stroke survivors living in the community, leading to a more holistic delivery of post-hospital rehabilitation care.

### Strengths and limitations

The TILDA dataset is a rich source of population health information, underpinned by a robust methodology. A major strength of this study is the large sample of nationally representative older adults. This study utilised the entire wave 1 cohort in order to compare healthcare utilisation between stroke and non-stroke participants, to establish the independent effects of stroke and cognitive function on healthcare utilisation. Cognitive function was measured using the MoCA, which is suitable for cognitively intact populations, and is more sensitive to the detection of mild cognitive impairment than the Mini-Mental State Examination (MMSE) [36]. However, it is important to note that cognitive screening tests are not diagnostic tools; rather, these assessments provide an indication of cognitive difficulties that require further investigation through comprehensive neuropsychological assessment. Hence, screening tests using standard cut-off values may fail to identify every individual with cognitive impairment. For this reason, the continuous MoCA score (converted to a standardised score) was utilised in the regression models, which increased the power of these analyses.

This study had several limitations. The number of stroke participants with a completed cognitive assessment was small (*n* = 92); hence, it was not appropriate to conduct multivariate regression analysis on the stroke sample specifically. In an effort to overcome this issue, the entire wave 1 sample was included in the regression models, and the relationship between stroke status and cognitive score was explored using an interaction test. However, the analysis may have lacked statistical power to identify interactions, due to the small number of participants in the stroke and cognitive impairment groups. In wave 1, less than half of participants with stroke (*n* = 51, 39%) had experienced stroke within the previous 3 years, and healthcare utilisation rates may differ significantly among those with less recent stroke. Limitations pertaining to the public TILDA dataset were also evident. Speech and language therapy was not available in this dataset for analysis, though the numbers using this service were extremely small (*n* = 19/8504) [66], and are unlikely to have changed the presented results. Additionally, the dataset did not include information on the reason for hospital re-admission or Emergency Department presentation, which would have influenced length of hospital stay and patient outcomes. It was not possible to adjust for a number of potential confounders; for example, this study did not explore the impact of non-cardiovascular comorbidities and chronic health conditions, which could increase healthcare utilisation in older populations [67]. Healthcare coverage was selected as a proxy for socioeconomic status in the regression analysis. Given that most of the Irish population over age 70 possess a medical card or GP visit card, a more specific measure of socioeconomic status would have been preferable.

Other limitations related to the generalisability of the findings. Firstly, healthcare utilisation in TILDA was self-reported, which raises the question of recall accuracy, especially among participants with cognitive impairment. Likewise, cognitive impairment can affect insight and reduce an individual’s awareness of their own health status, suggesting that self-reported diagnosis may be less accurate among those affected by cognitive decline. Selection bias is also a concern [68], in that cognitive data were only available from participants who took part in the TILDA health assessments, and these respondents tended to be younger and more able-bodied, with higher levels of education than the overall TILDA sample [69]. Thirdly, misclassification bias during data collection is also a possibility [68]; participants who experienced minor strokes or transient ischaemic attacks for which medical attention was never sought, may not have been classified appropriately (non-differential misclassification). Finally, nursing home residents and people with known or suspected dementia were excluded from wave 1 of TILDA [34]. Given these different potential sources of bias, the results of the present study may underestimate the prevalence of cognitive impairment. In addition, the utilisation rates reported in this study may underestimate health service use in more severe cases of stroke and cognitive impairment.

It was not possible to look at data in more recent waves of the public TILDA datasets as many of the required variables (e.g., healthcare utilisation, stroke status or cognitive status) were either not available, or were recoded as categorical variables, making comparison problematic. However, the results of this study are still relevant. Post-acute stroke care in Ireland has not changed significantly over the last decade [70], and access to rehabilitation continues to be an enduring problem for stroke survivors living in the community [29, 31, 64]. This study provides an indication of healthcare utilisation in stroke, and has identified poor cognitive function as a potential barrier to accessing outpatient services. These findings have important implications for the future planning of healthcare services for stroke survivors and individuals with cognitive impairment.

## Conclusion

This study investigated the effect of stroke, cognitive function and PSCI on healthcare utilisation in a nationally representative sample of community-dwelling older adults in Ireland. Stroke survivors exhibited significantly higher utilisation of GP and outpatient services, while poor cognitive function was associated with increased GP visits and fewer visits to outpatient services. These findings indicate that individuals with cognitive impairment may be underserved by outpatient healthcare services, particularly those with PSCI, which has important implications for healthcare provision. Identifying the healthcare services most frequently used by patients with stroke, cognitive impairment and PSCI will provide an opportunity for focused health service planning. Improved integration of services and the development of community stroke rehabilitation teams could facilitate a seamless transition of stroke patients between services, and promote timely access to post-stroke rehabilitation.

## Supplementary Information

**Additional file 1.** Regression tests with other covariates.

**Additional file 2:** **Table 1.** Demographic variables all comparisons.

**Additional file 3:** **Table 2.** HSU all comparisons.

**Additional file 4:** **Table 3.** Unadjusted and adjusted associations HSU.

## Data Availability

The public TILDA dataset supporting the conclusions of this article is available from the Irish Social Science Data Archive (ISSDA) at University College Dublin (66): http://www.ucd.ie/issda/data/tilda/ and the Interuniversity Consortium for Political and Social Research (ICPSR) at the University of Michigan: http://www.icpsr.umich.edu/icpsrweb/NACDA/studies/34315.

## Abbreviations

PSCI: Post-Stroke Cognitive Impairment
GP: General Practitioner
MoCA: Montreal Cognitive Assessment
MMSE: Mini-Mental State Examination
TILDA: The Irish Longitudinal Study on Ageing

## Footnotes

1 Outpatient services are delivered in the hospital, but do not require patient admission. Services include consultant visits, procedures, diagnostic tests (e.g., X-Ray, CT scan) and treatments, and patients must be referred by a GP, emergency department, public consultant or private healthcare provider to access these services. It should be noted that participants in this study may have received rehabilitation (e.g., physiotherapy) through hospital outpatient services, but it was not possible to quantify this. Therefore, in this study rehabilitation services were defined as publicly available therapies delivered in the community.

2 TILDA participants were asked to identify whether they had used any community care/ social services in the previous 12 months, including rehabilitation services (physiotherapy, occupational therapy and psychology). These are public services provided by the state, which are free for medical cardholders, and patients can access these services via the GP or self-referral through local health centres. Rehabilitation services delivered through community care are distinct from rehabilitation services provided through outpatient hospital services, where referral from the hospital team is required.
