# Supplementary material for: The impact of stroke, cognitive function and post-stroke cognitive impairment (PSCI) on healthcare utilisation in Ireland: a cross-sectional nationally representative study
Source: BMC Health Serv Res. 2022 Mar 29;22:414. doi: 10.1186/s12913-022-07837-2 (PMC8962254; doi:10.1186/s12913-022-07837-2)
Supplement: Supplementary file 1 — Additional file 1. Regression tests with other covariates. [file 12913_2022_7837_MOESM1_ESM.pdf]

File name: Additional File 1 Regression tests with other covariates

File format: PDF

Title of data: Regression tests with other covariates

Description of data: A number of covariates, which had negligible effects on the overall results, were excluded in the final regression models. This file presents these regression models, including the following covariates: (i) Cardiovascular (CVD) conditions (none/1 or more); (ii) Private health insurance (yes/no); (iii) Healthcare coverage (no cover, medical card/GP visit card only, private insurance only, dual cover); (iv) and long-term health problem (yes/no).

Regression tests with other covariates

1. Multiple regressions including CVD conditions (no CVD/1+) as covariate

```
. tab CVD_w1

              see notes |      Freq.     Percent        Cum.
------------------------+-----------------------------------
       no CVD conditions |      2,964       36.26       36.26
at least 1 CVD condition |      5,211       63.74      100.00
------------------------+-----------------------------------
                  Total |      8,175      100.00
```

1a.GP visits - original model

```
. nbreg gpvisits i.strokew1 zMoca age i.sex i.edu3 i.emp2 i.medcard i.Disability2 MHcesd_capi,
irr nolog

Negative binomial regression                    Number of obs     =      5,753
                                                LR chi2(10)       =    1364.13
Dispersion     = mean                           Prob > chi2       =     0.0000
Log likelihood = -13146.315                     Pseudo R2         =     0.0493

------------------------------------------------------------------------------------
             gpvisits |        IRR   Std. Err.      z    P>|z|     [95% Conf. Interval]
----------------------+-------------------------------------------------------------
             strokew1 |
              stroke  |    1.26931   .1088749     2.78   0.005     1.072892    1.501687
                zMoca |   1.066888   .0136849     5.05   0.000     1.040401     1.09405
                  age |   1.003391   .0016149     2.10   0.035     1.000231    1.006561
                      |
                  sex |
              Female  |   1.016083   .0235785     0.69   0.492     .9709047    1.063363
                      |
                 edu3 |
        secondary edu |   .9953264   .0292704    -0.16   0.873     .9395795    1.054381
      third level edu |   .9537152   .0318513    -1.42   0.156     .8932871    1.018231
                      |
                 emp2 |
            Employed  |   .8489824   .0245915    -5.65   0.000     .8021266    .8985752
                      |
              medcard |
medcard/gp visit card |   1.597204   .0444586    16.82   0.000     1.512401    1.686762
                      |
          Disability2 |
```

```
          Disability  |   1.434818    .050219    10.32   0.000     1.339691      1.5367
          MHcesd_capi |   1.018779   .0016563    11.44   0.000     1.015538     1.02203
                _cons |   2.029435    .225706     6.36   0.000     1.631953    2.523728
----------------------+----------------------------------------------------------------
             /lnalpha |  -.8281074   .0318193                     -.8904721   -.7657428
----------------------+----------------------------------------------------------------
                alpha |   .4368753   .0139011                      .4104619    .4649884
---------------------------------------------------------------------------------------
LR test of alpha=0: chibar2(01) = 4075.71             Prob >= chibar2 = 0.000
```

1b.GP visits - model including CVD conditions

```
. nbreg gpvisits i.strokew1 zMoca i.CVD_w1 age i.sex i.edu3 i.emp2 i.medcard i.Disability2
MHcesd_capi, irr nolog

Negative binomial regression                    Number of obs     =      5,753
                                                LR chi2(11)       =    1576.37
Dispersion     = mean                           Prob > chi2       =     0.0000
Log likelihood = -13040.195                     Pseudo R2         =     0.0570

------------------------------------------------------------------------------------------
                 gpvisits |        IRR   Std. Err.      z    P>|z|     [95% Conf. Interval]
-------------------------+----------------------------------------------------------------
                 strokew1 |
                  stroke  |   1.230329   .1033133     2.47   0.014     1.043624    1.450436
                    zMoca |   1.075884   .0135795     5.79   0.000     1.049595    1.102831
                          |
                   CVD_w1 |
at least 1 CVD condition  |   1.438804   .0355696    14.72   0.000     1.370751    1.510236
                      age |    1.00019   .0016001     0.12   0.906     .9970587    1.003331
                          |
                      sex |
                  Female  |   1.024435   .0234327     1.06   0.291     .9795225    1.071408
                          |
                     edu3 |
           secondary edu  |   1.003648   .0290498     0.13   0.900     .9482965    1.062231
         third level edu  |   .9615744   .0316433    -1.19   0.234     .9015125    1.025638
                          |
                     emp2 |
                Employed  |   .8580756   .0245463    -5.35   0.000     .8112896    .9075596
                          |
                  medcard |
   medcard/gp visit card  |    1.57704   .0433139    16.59   0.000     1.494391    1.664261
                          |
              Disability2 |
              Disability  |   1.393207    .047925     9.64   0.000     1.302372    1.490377
              MHcesd_capi |   1.018333   .0016222    11.40   0.000     1.015159    1.021518
                    _cons |   1.932588   .2117988     6.01   0.000     1.559026    2.395661
-------------------------+----------------------------------------------------------------
                 /lnalpha |  -.8881818   .0325212                     -.9519223   -.8244413
-------------------------+----------------------------------------------------------------
                    alpha |   .4114031   .0133793                      .3859983    .4384799
------------------------------------------------------------------------------------------
LR test of alpha=0: chibar2(01) = 3800.37             Prob >= chibar2 = 0.000
```

2a.Emergency visits - original model

```
. nbreg emergency i.strokew1 zMoca age i.sex i.edu3 i.emp2 i.medcard i.Disability2 MHcesd_capi,
irr nolog

Negative binomial regression                    Number of obs     =      5,757
                                                LR chi2(10)       =     136.19
Dispersion     = mean                           Prob > chi2       =     0.0000
Log likelihood = -3156.5347                     Pseudo R2         =     0.0211

----------------------------------------------------------------------------------------
             emergency |        IRR   Std. Err.      z    P>|z|     [95% Conf. Interval]
-----------------------+----------------------------------------------------------------
              strokew1 |
               stroke  |   1.562992   .4085484     1.71   0.088      .936402    2.608863
                 zMoca |   1.055994   .0455497     1.26   0.207     .9703874    1.149152
                   age |   1.000074   .0053157     0.01   0.989     .9897096    1.010547
                       |
                   sex |
               Female  |   .9694865   .0738441    -0.41   0.684       .83504     1.12558
                       |
                  edu3 |
        secondary edu  |    .981302   .0952215    -0.19   0.846      .811345    1.186861
      third level edu  |   1.150641   .1256091     1.29   0.199     .9290065     1.42515
                       |
                  emp2 |
             Employed  |   .9343425   .0892415    -0.71   0.477     .7748286    1.126696
                       |
               medcard |
medcard/gp visit card  |   1.425844   .1318271     3.84   0.000     1.189525    1.709111
                       |
           Disability2 |
           Disability  |   1.880549   .1998814     5.94   0.000     1.526903    2.316103
           MHcesd_capi |   1.021914   .0050527     4.38   0.000     1.012059    1.031865
                 _cons |   .1416426   .0518411    -5.34   0.000     .0691282    .2902237
-----------------------+----------------------------------------------------------------
              /lnalpha |   1.135652    .078928                      .9809563    1.290348
-----------------------+----------------------------------------------------------------
                 alpha |   3.113204   .2457189                      2.667005    3.634052
----------------------------------------------------------------------------------------
LR test of alpha=0: chibar2(01) = 644.50              Prob >= chibar2 = 0.000
```

2b.Emergency visits - model including CVD conditions

```
. nbreg emergency i.strokew1 zMoca i.CVD_w1 age i.sex i.edu3 i.emp2 i.medcard i.Disability2
MHcesd_capi, irr nolog

Negative binomial regression                    Number of obs     =      5,757
                                                LR chi2(11)       =     142.77
Dispersion     = mean                           Prob > chi2       =     0.0000
Log likelihood = -3153.2471                     Pseudo R2         =     0.0221

------------------------------------------------------------------------------------------
               emergency |        IRR   Std. Err.      z    P>|z|     [95% Conf. Interval]
-------------------------+----------------------------------------------------------------
                strokew1 |
                  stroke  |   1.529209   .3990983     1.63   0.104     .9168897    2.550447
                   zMoca |   1.060109   .0457456     1.35   0.176     .9741362    1.153669
                         |
                  CVD_w1 |
at least 1 CVD condition  |   1.235639   .1021309     2.56   0.010     1.050839    1.452938
                     age |   .9980633   .0053591    -0.36   0.718     .9876147    1.008622
                         |
                     sex |
                  Female  |   .9690903    .073761    -0.41   0.680      .834788       1.125
                         |
                    edu3 |
           secondary edu  |    .983699   .0953829    -0.17   0.865     .8134424    1.189591
         third level edu  |   1.147465   .1251908     1.26   0.207     .9265562    1.421043
                         |
                    emp2 |
                Employed  |   .9394261    .089723    -0.65   0.513     .7790508    1.132816
                         |
                 medcard |
   medcard/gp visit card  |   1.418072   .1310527     3.78   0.000     1.183133    1.699664
                         |
             Disability2 |
              Disability  |   1.835207    .195486     5.70   0.000     1.489414    2.261284
             MHcesd_capi |   1.021596   .0050429     4.33   0.000      1.01176    1.031528
                   _cons |   .1404339    .051381    -5.37   0.000     .0685551    .2876763
-------------------------+----------------------------------------------------------------
                /lnalpha |   1.127702   .0791117                      .9726459    1.282758
-------------------------+----------------------------------------------------------------
                   alpha |   3.088551   .2443406                      2.644933    3.606574
------------------------------------------------------------------------------------------
LR test of alpha=0: chibar2(01) = 639.56              Prob >= chibar2 = 0.000
```

3a.Nights in hosptal - original model

```
. nbreg nights i.strokew1 zMoca age i.sex i.edu3 i.emp2 i.medcard i.Disability2 MHcesd_capi, irr
nolog

Negative binomial regression                    Number of obs     =      5,758
                                                LR chi2(10)       =     100.85
Dispersion     = mean                           Prob > chi2       =     0.0000
Log likelihood = -4043.7539                     Pseudo R2         =     0.0123

------------------------------------------------------------------------------------
              nights |        IRR   Std. Err.      z    P>|z|     [95% Conf. Interval]
---------------------+--------------------------------------------------------------
            strokew1 |
             stroke  |   1.930541   .8994416     1.41   0.158     .7746455    4.811217
               zMoca |   1.047743   .0662378     0.74   0.461       .92564    1.185952
                 age |   1.021044   .0088188     2.41   0.016     1.003905    1.038475
                     |
                 sex |
             Female  |   .9332921   .1110405    -0.58   0.562     .7391694    1.178396
                     |
                edu3 |
       secondary edu |   1.233323   .1891828     1.37   0.172     .9130801    1.665885
     third level edu |   1.251192   .2208183     1.27   0.204     .8853166    1.768274
                     |
                emp2 |
           Employed  |   .7813591   .1203808    -1.60   0.109     .5777096    1.056798
                     |
             medcard |
medcard/gp visit card |   1.518647   .2199657     2.88   0.004     1.143315    2.017193
                     |
         Disability2 |
         Disability  |   2.106449   .3963532     3.96   0.000     1.456758    3.045892
         MHcesd_capi |     1.0189   .0088877     2.15   0.032     1.001628    1.036469
               _cons |   .0973825   .0587259    -3.86   0.000     .0298655    .3175357
---------------------+--------------------------------------------------------------
            /lnalpha |   2.852873   .0487549                      2.757315    2.948431
---------------------+--------------------------------------------------------------
               alpha |   17.33752   .8452886                      15.75748    19.07599
------------------------------------------------------------------------------------
LR test of alpha=0: chibar2(01) = 1.0e+04              Prob >= chibar2 = 0.000
```

3b.Nights in hospital - model including CVD conditions

```
. nbreg nights i.strokew1 zMoca i.CVD_w1 age i.sex i.edu3 i.emp2 i.medcard i.Disability2
MHcesd_capi, irr nolog

Negative binomial regression                    Number of obs     =      5,758
                                                LR chi2(11)       =     108.87
Dispersion     = mean                           Prob > chi2       =     0.0000
Log likelihood = -4039.7446                     Pseudo R2         =     0.0133

--------------------------------------------------------------------------------------------
                 nights |        IRR   Std. Err.      z    P>|z|     [95% Conf. Interval]
-------------------------+------------------------------------------------------------------
               strokew1 |
                stroke  |   1.982573   .9217095     1.47   0.141     .7970781    4.931257
                  zMoca |   1.062547   .0670968     0.96   0.337      .938852    1.202539
                        |
                 CVD_w1 |
at least 1 CVD condition  |   1.442755   .1839679     2.87   0.004     1.123711    1.852382
                    age |   1.017069    .008892     1.94   0.053     .9997894    1.034647
                        |
                    sex |
                Female  |   .9446874   .1122316    -0.48   0.632     .7484502    1.192376
                        |
                   edu3 |
         secondary edu  |   1.267437   .1946099     1.54   0.123     .9380539    1.712478
       third level edu  |   1.229091   .2161316     1.17   0.241     .8707695    1.734863
                        |
                   emp2 |
              Employed  |   .7880019   .1214622    -1.55   0.122     .5825371    1.065936
                        |
                medcard |
   medcard/gp visit card  |   1.476801   .2136654     2.69   0.007     1.112165    1.960988
                        |
            Disability2 |
            Disability  |   2.023287   .3807571     3.74   0.000     1.399175    2.925788
            MHcesd_capi |    1.01768   .0088037     2.03   0.043      1.00057    1.035082
                  _cons |   .0978585     .05891    -3.86   0.000     .0300734    .3184304
-------------------------+------------------------------------------------------------------
               /lnalpha |   2.844353   .0488406                      2.748627    2.940079
-------------------------+------------------------------------------------------------------
                  alpha |   17.19043   .8395916                      15.62116    18.91733
--------------------------------------------------------------------------------------------
LR test of alpha=0: chibar2(01) = 1.0e+04              Prob >= chibar2 = 0.000
```

4a.Outpatient visits - original model

```
. nbreg outpatient i.strokew1 zMoca age i.sex i.edu3 i.emp2 i.medcard i.Disability2 MHcesd_capi,
irr nolog

Negative binomial regression                    Number of obs     =      5,758
                                                LR chi2(10)       =     320.19
Dispersion     = mean                           Prob > chi2       =     0.0000
Log likelihood = -8323.6887                     Pseudo R2         =     0.0189

----------------------------------------------------------------------------------------
           outpatient |        IRR   Std. Err.      z    P>|z|     [95% Conf. Interval]
----------------------+-----------------------------------------------------------------
             strokew1 |
              stroke  |   1.491691   .2666236     2.24   0.025     1.050838    2.117493
                zMoca |   .9233992   .0251754    -2.92   0.003     .8753515    .9740841
                  age |   .9991229   .0033752    -0.26   0.795     .9925296     1.00576
                      |
                  sex |
              Female  |    .970144   .0461093    -0.64   0.524      .883853     1.06486
                      |
                 edu3 |
       secondary edu  |   .9581131   .0588409    -0.70   0.486     .8494575    1.080667
     third level edu  |   1.048307    .072027     0.69   0.492     .9162296    1.199424
                      |
                 emp2 |
            Employed  |   .6883726   .0408307    -6.30   0.000     .6128225    .7732367
                      |
              medcard |
medcard/gp visit card  |   1.308491   .0735854     4.78   0.000      1.17193    1.460964
                      |
          Disability2 |
          Disability  |   1.786977     .12974     8.00   0.000     1.549955    2.060244
          MHcesd_capi |   1.021031   .0035114     6.05   0.000     1.014172    1.027936
                _cons |   1.027715   .2380878     0.12   0.906     .6526458    1.618334
----------------------+-----------------------------------------------------------------
             /lnalpha |   .7918733   .0349612                      .7233506     .860396
----------------------+-----------------------------------------------------------------
                alpha |   2.207528   .0771779                      2.061328    2.364097
----------------------------------------------------------------------------------------
LR test of alpha=0: chibar2(01) = 5125.52              Prob >= chibar2 = 0.000
```

4b.Outpatient visits - model including CVD conditions

```
. nbreg outpatient i.strokew1 zMoca i.CVD_w1 age i.sex i.edu3 i.emp2 i.medcard i.Disability2
MHcesd_capi, irr nolog

Negative binomial regression                    Number of obs     =      5,758
                                                LR chi2(11)       =     371.72
Dispersion     = mean                           Prob > chi2       =     0.0000
Log likelihood = -8297.9233                     Pseudo R2         =     0.0219

------------------------------------------------------------------------------------------
              outpatient |        IRR   Std. Err.      z    P>|z|     [95% Conf. Interval]
-------------------------+----------------------------------------------------------------
                strokew1 |
                 stroke  |   1.416524   .2513117     1.96   0.050      1.00048    2.005576
                   zMoca |   .9333502   .0252937    -2.55   0.011      .885069    .9842652
                         |
                  CVD_w1 |
at least 1 CVD condition |   1.448863   .0740592     7.25   0.000     1.310743    1.601536
                     age |   .9953236   .0033865    -1.38   0.168     .9887082    1.001983
                         |
                     sex |
                 Female  |   .9818089   .0464801    -0.39   0.698     .8948083    1.077268
                         |
                    edu3 |
          secondary edu  |    .963217   .0588214    -0.61   0.539     .8545614    1.085688
        third level edu  |   1.057727   .0722966     0.82   0.412     .9251093    1.209355
                         |
                    emp2 |
               Employed  |   .7035111   .0416331    -5.94   0.000     .6264663    .7900312
                         |
                 medcard |
   medcard/gp visit card |   1.306792   .0731684     4.78   0.000     1.170973    1.458364
                         |
             Disability2 |
             Disability  |   1.732607   .1251426     7.61   0.000     1.503902    1.996092
             MHcesd_capi |   1.020224   .0034795     5.87   0.000     1.013427    1.027067
                   _cons |   1.004379   .2314683     0.02   0.985     .6393376    1.577846
-------------------------+----------------------------------------------------------------
                /lnalpha |   .7695325   .0352299                      .7004832    .8385819
-------------------------+----------------------------------------------------------------
                   alpha |   2.158757   .0760528                      2.014726    2.313085
------------------------------------------------------------------------------------------
LR test of alpha=0: chibar2(01) = 4994.43             Prob >= chibar2 = 0.000
```

5a.Rehab services used - 3 services (new variable: OT, Psychology and Physiotherapy)

```
. logit SSused3 i.strokew1 zMoca age i.sex i.edu3 i.emp2 i.medcard i.Disability2 MHcesd_capi, or
nolog

Logistic regression                              Number of obs     =      5,760
                                                 LR chi2(10)       =     260.77
                                                 Prob > chi2       =     0.0000
Log likelihood = -1259.2078                      Pseudo R2         =     0.0938

-------------------------------------------------------------------------------------
              SSused3 | Odds Ratio   Std. Err.      z    P>|z|     [95% Conf. Interval]
----------------------+--------------------------------------------------------------
             strokew1 |
              stroke  |   1.247889   .4231788     0.65   0.514      .641978     2.42567
                zMoca |   .9854331   .0581176    -0.25   0.804     .8778617    1.106186
                  age |   .9876777   .0072565    -1.69   0.091     .9735571    1.002003
                      |
                  sex |
              Female  |   1.139399    .129593     1.15   0.251     .9117204    1.423934
                      |
                 edu3 |
       secondary edu  |   1.447394   .2076175     2.58   0.010     1.092668    1.917278
     third level edu  |   1.763759   .2863017     3.50   0.000     1.283122    2.424433
                      |
                 emp2 |
            Employed  |   .6002524   .0949352    -3.23   0.001     .4402596    .8183875
                      |
              medcard |
medcard/gp visit card |   2.697211   .3800268     7.04   0.000     2.046369    3.555053
                      |
          Disability2 |
          Disability  |   2.593357   .3499073     7.06   0.000     1.990739    3.378394
          MHcesd_capi |   1.039322   .0066217     6.05   0.000     1.026425    1.052382
                _cons |   .0424572   .0217686    -6.16   0.000     .0155426    .1159789
-------------------------------------------------------------------------------------
```

5b. Rehab services used (3 services) - model including CVD conditions

```
. logit SSused3 i.strokew1 zMoca i.CVD_w1 age i.sex i.edu3 i.emp2 i.medcard i.Disability2
MHcesd_capi, or nolog

Logistic regression                             Number of obs     =      5,760
                                                LR chi2(11)       =     262.96
                                                Prob > chi2       =     0.0000
Log likelihood = -1258.1131                     Pseudo R2         =     0.0946

------------------------------------------------------------------------------------------
                  SSused3 | Odds Ratio   Std. Err.      z    P>|z|     [95% Conf. Interval]
-------------------------+----------------------------------------------------------------
                 strokew1 |
                  stroke  |   1.229696   .4163436     0.61   0.541       .63329    2.387772
                    zMoca |   .9895971   .0585067    -0.18   0.860     .8813205    1.111176
                          |
                   CVD_w1 |
at least 1 CVD condition  |    1.20336   .1519055     1.47   0.143     .9396035    1.541156
                      age |   .9860614   .0073258    -1.89   0.059      .971807    1.000525
                          |
                      sex |
                  Female  |   1.141191   .1298287     1.16   0.246     .9131051    1.426252
                          |
                     edu3 |
           secondary edu  |   1.456619   .2090895     2.62   0.009     1.099413    1.929884
         third level edu  |   1.774354   .2882235     3.53   0.000     1.290542    2.439541
                          |
                     emp2 |
                Employed  |   .6039991   .0955388    -3.19   0.001     .4429918    .8235252
                          |
                  medcard |
   medcard/gp visit card  |   2.686256   .3784398     7.01   0.000     2.038122    3.540501
                          |
              Disability2 |
              Disability  |    2.54289   .3445207     6.89   0.000      1.94986    3.316284
              MHcesd_capi |   1.039224   .0066269     6.03   0.000     1.026316    1.052294
                    _cons |    .041506   .0213053    -6.20   0.000      .015177    .1135108
------------------------------------------------------------------------------------------
```

6a.Rehab services used - 2 services only (original variable: OT and Psychology)

```
. logit RehabUsed2 i.strokew1 zMoca age i.sex i.edu3 i.emp2 i.medcard i.Disability2 MHcesd_capi,
or nolog

Logistic regression                             Number of obs     =      5,760
                                                LR chi2(10)       =     160.85
                                                Prob > chi2       =     0.0000
Log likelihood = -533.39536                     Pseudo R2         =     0.1310

---------------------------------------------------------------------------------------
           RehabUsed2 | Odds Ratio   Std. Err.      z    P>|z|     [95% Conf. Interval]
----------------------+----------------------------------------------------------------
             strokew1 |
              stroke  |   3.023982   1.210921     2.76   0.006      1.37951    6.628779
                zMoca |   .9380939   .0918678    -0.65   0.514     .7742619    1.136592
                  age |   .9660501   .0117159    -2.85   0.004     .9433582    .9892879
                      |
                  sex |
              Female  |     .98947   .1873641    -0.06   0.955     .6826857    1.434117
                      |
                 edu3 |
       secondary edu  |   1.573118   .3827416     1.86   0.063     .9764793     2.53431
     third level edu  |   2.043387   .5582288     2.62   0.009     1.196224    3.490509
                      |
                 emp2 |
            Employed  |   .4544699   .1282731    -2.79   0.005     .2613698    .7902326
                      |
              medcard |
medcard/gp visit card |   2.865971   .6841755     4.41   0.000     1.795022    4.575872
                      |
          Disability2 |
          Disability  |   3.386041   .7127287     5.79   0.000     2.241416    5.115191
          MHcesd_capi |   1.048094   .0098247     5.01   0.000     1.029013    1.067528
                _cons |   .0442429   .0373463    -3.69   0.000     .0084591    .2313985
---------------------------------------------------------------------------------------
```

6b. Rehab services used (2 services) - model including CVD conditions

```
. logit RehabUsed2 i.strokew1 zMoca i.CVD_w1 age i.sex i.edu3 i.emp2 i.medcard i.Disability2
MHcesd_capi, or nolog

Logistic regression                              Number of obs     =      5,760
                                                 LR chi2(11)       =     161.40
                                                 Prob > chi2       =     0.0000
Log likelihood = -533.12063                      Pseudo R2         =     0.1315

---------------------------------------------------------------------------------------------
               RehabUsed2 | Odds Ratio   Std. Err.      z    P>|z|     [95% Conf. Interval]
--------------------------+------------------------------------------------------------------
                 strokew1 |
                  stroke  |   2.985287   1.194223     2.73   0.006     1.362933    6.538794
                    zMoca |    .942024   .0924501    -0.61   0.543     .7771863    1.141823
                          |
                   CVD_w1 |
at least 1 CVD condition  |    1.16932   .2488205     0.74   0.462     .7705582    1.774439
                      age |   .9648015   .0118178    -2.93   0.003     .9419149    .9882441
                          |
                      sex |
                  Female  |   .9907469   .1876009    -0.05   0.961     .6835734    1.435953
                          |
                     edu3 |
           secondary edu  |   1.586559   .3866118     1.89   0.058     .9840929    2.557859
         third level edu  |   2.057371   .5626951     2.64   0.008      1.20367    3.516559
                          |
                     emp2 |
                Employed  |   .4577112   .1292737    -2.77   0.006     .2631372    .7961607
                          |
                  medcard |
   medcard/gp visit card  |   2.857973   .6822748     4.40   0.000     1.790002    4.563129
                          |
              Disability2 |
              Disability  |   3.322225   .7037876     5.67   0.000     2.193354    5.032103
              MHcesd_capi |   1.048187   .0098382     5.01   0.000      1.02908    1.067648
                    _cons |   .0429982   .0363596    -3.72   0.000     .0081972    .2255449
---------------------------------------------------------------------------------------------
```

****

2. Multiple regressions including other covariates

Insurance (ins2)

```
. tab ins2

               ins2 |      Freq.     Percent        Cum.
--------------------+-----------------------------------
no private insurance |      3,465       42.42       42.42
   private insurance |      4,704       57.58      100.00
--------------------+-----------------------------------
               Total |      8,169      100.00
```

1a.GP visits - original model

```
. nbreg gpvisits i.strokew1 zMoca age i.sex i.edu3 i.emp2 i.medcard i.Disability2 MHcesd_capi,
irr nolog

Negative binomial regression                    Number of obs     =      5,753
                                                LR chi2(10)       =    1364.13
Dispersion     = mean                           Prob > chi2       =     0.0000
Log likelihood = -13146.315                     Pseudo R2         =     0.0493

-------------------------------------------------------------------------------------
             gpvisits |        IRR   Std. Err.      z    P>|z|     [95% Conf. Interval]
----------------------+--------------------------------------------------------------
             strokew1 |
              stroke  |    1.26931   .1088749     2.78   0.005     1.072892    1.501687
                zMoca |   1.066888   .0136849     5.05   0.000     1.040401     1.09405
                  age |   1.003391   .0016149     2.10   0.035     1.000231    1.006561
                      |
                  sex |
               Female  |   1.016083   .0235785     0.69   0.492     .9709047    1.063363
                      |
                 edu3 |
        secondary edu  |   .9953264   .0292704    -0.16   0.873     .9395795    1.054381
      third level edu  |   .9537152   .0318513    -1.42   0.156     .8932871    1.018231
                      |
                 emp2 |
             Employed  |   .8489824   .0245915    -5.65   0.000     .8021266    .8985752
                      |
              medcard |
medcard/gp visit card  |   1.597204   .0444586    16.82   0.000     1.512401    1.686762
                      |
          Disability2 |
           Disability  |   1.434818    .050219    10.32   0.000     1.339691      1.5367
          MHcesd_capi |   1.018779   .0016563    11.44   0.000     1.015538     1.02203
```

```
               _cons |   2.029435    .225706     6.36   0.000     1.631953    2.523728
---------------------+----------------------------------------------------------------
            /lnalpha |  -.8281074   .0318193                     -.8904721   -.7657428
---------------------+----------------------------------------------------------------
               alpha |   .4368753   .0139011                      .4104619    .4649884
--------------------------------------------------------------------------------------
LR test of alpha=0: chibar2(01) = 4075.71             Prob >= chibar2 = 0.000
```

1b.GP visits - model including insurance

```
. nbreg gpvisits i.strokew1 zMoca i.ins2 age i.sex i.edu3 i.emp2 i.medcard i.Disability2
MHcesd_capi, irr nolog

Negative binomial regression                    Number of obs     =      5,749
                                                LR chi2(11)       =    1353.30
Dispersion     = mean                           Prob > chi2       =     0.0000
Log likelihood = -13137.446                     Pseudo R2         =     0.0490

------------------------------------------------------------------------------------
            gpvisits |        IRR   Std. Err.      z    P>|z|     [95% Conf. Interval]
---------------------+--------------------------------------------------------------
            strokew1 |
             stroke  |   1.269977   .1089374     2.79   0.005     1.073447    1.502488
               zMoca |   1.066563   .0137304     5.01   0.000     1.039989    1.093817
                     |
                ins2 |
   private insurance |   .9964236   .0275354    -0.13   0.897     .9438907     1.05188
                 age |   1.003471   .0016595     2.10   0.036     1.000224    1.006729
                     |
                 sex |
             Female  |   1.015687   .0235792     0.67   0.503     .9705083    1.062969
                     |
                edu3 |
       secondary edu |   .9949325   .0298201    -0.17   0.865     .9381697     1.05513
     third level edu |   .9541238   .0327592    -1.37   0.171     .8920296     1.02054
                     |
                emp2 |
           Employed  |   .8491743    .024612    -5.64   0.000     .8022803    .8988093
                     |
             medcard |
medcard/gp visit card |  1.593726    .048097    15.44   0.000     1.502192    1.690838
                     |
         Disability2 |
          Disability |   1.432837   .0502106    10.26   0.000      1.33773    1.534706
         MHcesd_capi |   1.018631   .0016614    11.32   0.000      1.01538    1.021893
               _cons |   2.028307   .2259408     6.35   0.000     1.630478    2.523203
---------------------+--------------------------------------------------------------
            /lnalpha |  -.8277287   .0318298                     -.8901141   -.7653434
---------------------+--------------------------------------------------------------
               alpha |   .4370408   .0139109                      .4106089    .4651742
------------------------------------------------------------------------------------
LR test of alpha=0: chibar2(01) = 4070.53             Prob >= chibar2 = 0.000
```

2a.Emergency visits - original model

```
. nbreg emergency i.strokew1 zMoca age i.sex i.edu3 i.emp2 i.medcard i.Disability2 MHcesd_capi,
irr nolog

Negative binomial regression                    Number of obs     =      5,757
                                                LR chi2(10)       =     136.19
Dispersion     = mean                           Prob > chi2       =     0.0000
Log likelihood = -3156.5347                     Pseudo R2         =     0.0211

------------------------------------------------------------------------------------
             emergency |        IRR   Std. Err.      z    P>|z|     [95% Conf. Interval]
----------------------+-------------------------------------------------------------
             strokew1 |
               stroke  |   1.562992   .4085484     1.71   0.088      .936402    2.608863
                zMoca |   1.055994   .0455497     1.26   0.207     .9703874    1.149152
                  age |   1.000074   .0053157     0.01   0.989     .9897096    1.010547
                      |
                  sex |
               Female  |   .9694865   .0738441    -0.41   0.684       .83504     1.12558
                      |
                 edu3 |
        secondary edu  |    .981302   .0952215    -0.19   0.846      .811345    1.186861
      third level edu  |   1.150641   .1256091     1.29   0.199     .9290065     1.42515
                      |
                 emp2 |
             Employed  |   .9343425   .0892415    -0.71   0.477     .7748286    1.126696
                      |
              medcard |
medcard/gp visit card  |   1.425844   .1318271     3.84   0.000     1.189525    1.709111
                      |
          Disability2 |
           Disability  |   1.880549   .1998814     5.94   0.000     1.526903    2.316103
          MHcesd_capi |   1.021914   .0050527     4.38   0.000     1.012059    1.031865
                _cons |   .1416426   .0518411    -5.34   0.000     .0691282    .2902237
----------------------+-------------------------------------------------------------
             /lnalpha |   1.135652    .078928                       .9809563    1.290348
----------------------+-------------------------------------------------------------
                alpha |   3.113204   .2457189                       2.667005    3.634052
------------------------------------------------------------------------------------
LR test of alpha=0: chibar2(01) = 644.50              Prob >= chibar2 = 0.000
```

2b. Emergency visits - model including insurance

```
. nbreg emergency i.strokew1 zMoca i.ins2 age i.sex i.edu3 i.emp2 i.medcard i.Disability2
MHcesd_capi, irr nolog

Negative binomial regression                    Number of obs     =      5,753
                                                LR chi2(11)       =     132.49
Dispersion     = mean                           Prob > chi2       =     0.0000
Log likelihood = -3150.4197                     Pseudo R2         =     0.0206

------------------------------------------------------------------------------------
          emergency |        IRR   Std. Err.      z    P>|z|     [95% Conf. Interval]
----------------------+-------------------------------------------------------------
           strokew1 |
             stroke  |   1.559522   .4062789     1.71   0.088     .9359244    2.598617
              zMoca |   1.049293   .0453461     1.11   0.266      .964076    1.142042
                    |
               ins2 |
    private insurance  |   .8688459   .0777711    -1.57   0.116     .7290395    1.035463
                age |   1.002204     .00546     0.40   0.686     .9915593    1.012962
                    |
                sex |
             Female  |   .9707361   .0739303    -0.39   0.697     .8361313     1.12701
                    |
               edu3 |
      secondary edu  |   1.006462   .0999081     0.06   0.948      .828517    1.222625
    third level edu  |    1.19967   .1353425     1.61   0.107     .9616836     1.49655
                    |
               emp2 |
           Employed  |   .9359685   .0893195    -0.69   0.488     .7763027    1.128474
                    |
            medcard |
medcard/gp visit card  |   1.344173   .1329223     2.99   0.003     1.107342    1.631656
                    |
        Disability2 |
         Disability  |   1.846807   .1966862     5.76   0.000     1.498884    2.275491
        MHcesd_capi |   1.020814   .0050905     4.13   0.000     1.010885     1.03084
              _cons |   .1364452   .0499853    -5.44   0.000     .0665471     .279761
----------------------+-------------------------------------------------------------
           /lnalpha |   1.131686    .079167                       .9765215     1.28685
----------------------+-------------------------------------------------------------
              alpha |    3.10088   .2454874                       2.655204    3.621363
------------------------------------------------------------------------------------
LR test of alpha=0: chibar2(01) = 635.78              Prob >= chibar2 = 0.000
```

3a.Nights in hospital - original model

```
. nbreg nights i.strokew1 zMoca age i.sex i.edu3 i.emp2 i.medcard i.Disability2 MHcesd_capi, irr
nolog

Negative binomial regression                    Number of obs     =      5,758
                                                LR chi2(10)       =     100.85
Dispersion     = mean                           Prob > chi2       =     0.0000
Log likelihood = -4043.7539                     Pseudo R2         =     0.0123

------------------------------------------------------------------------------------
               nights |        IRR   Std. Err.      z    P>|z|     [95% Conf. Interval]
----------------------+-------------------------------------------------------------
             strokew1 |
              stroke  |   1.930541   .8994416     1.41   0.158     .7746455    4.811217
                zMoca |   1.047743   .0662378     0.74   0.461       .92564    1.185952
                  age |   1.021044   .0088188     2.41   0.016     1.003905    1.038475
                      |
                  sex |
              Female  |   .9332921   .1110405    -0.58   0.562     .7391694    1.178396
                      |
                 edu3 |
        secondary edu |   1.233323   .1891828     1.37   0.172     .9130801    1.665885
      third level edu |   1.251192   .2208183     1.27   0.204     .8853166    1.768274
                      |
                 emp2 |
            Employed  |   .7813591   .1203808    -1.60   0.109     .5777096    1.056798
                      |
              medcard |
medcard/gp visit card |   1.518647   .2199657     2.88   0.004     1.143315    2.017193
                      |
          Disability2 |
          Disability  |   2.106449   .3963532     3.96   0.000     1.456758    3.045892
          MHcesd_capi |     1.0189   .0088877     2.15   0.032     1.001628    1.036469
                _cons |   .0973825   .0587259    -3.86   0.000     .0298655    .3175357
----------------------+-------------------------------------------------------------
             /lnalpha |   2.852873   .0487549                      2.757315    2.948431
----------------------+-------------------------------------------------------------
                alpha |   17.33752   .8452886                      15.75748    19.07599
------------------------------------------------------------------------------------
LR test of alpha=0: chibar2(01) = 1.0e+04             Prob >= chibar2 = 0.000
```

3b.Nights in hospital - model including insurance

```
. nbreg nights i.strokew1 zMoca i.ins2 age i.sex i.edu3 i.emp2 i.medcard i.Disability2
MHcesd_capi, irr nolog

Negative binomial regression                    Number of obs     =      5,754
                                                LR chi2(11)       =     100.77
Dispersion     = mean                           Prob > chi2       =     0.0000
Log likelihood = -4040.2153                     Pseudo R2         =     0.0123

------------------------------------------------------------------------------------
               nights |        IRR   Std. Err.      z    P>|z|     [95% Conf. Interval]
----------------------+-------------------------------------------------------------
             strokew1 |
              stroke  |    1.92784   .8988453     1.41   0.159     .7730417    4.807721
                zMoca |   1.048222   .0663228     0.74   0.457     .9259687    1.186616
                      |
                 ins2 |
    private insurance  |   1.070257   .1617246     0.45   0.653     .7959114    1.439169
                  age |   1.020357   .0089522     2.30   0.022     1.002961    1.038055
                      |
                  sex |
              Female  |   .9286919   .1109297    -0.62   0.536     .7348487    1.173668
                      |
                 edu3 |
        secondary edu  |   1.209907   .1927329     1.20   0.232     .8854404    1.653274
      third level edu  |   1.221389   .2252715     1.08   0.278     .8508625    1.753269
                      |
                 emp2 |
            Employed  |   .7838596   .1209826    -1.58   0.115     .5792445    1.060754
                      |
              medcard |
medcard/gp visit card  |   1.566816   .2534868     2.78   0.006     1.141056    2.151441
                      |
          Disability2 |
          Disability  |   2.116934   .3993409     3.98   0.000     1.462634    3.063931
          MHcesd_capi |   1.019098   .0089096     2.16   0.030     1.001785    1.036711
                _cons |   .0974991   .0588243    -3.86   0.000     .0298843    .3180956
----------------------+-------------------------------------------------------------
             /lnalpha |   2.854183   .0487796                      2.758577    2.949789
----------------------+-------------------------------------------------------------
                alpha |   17.36025   .8468264                      15.77737    19.10193
------------------------------------------------------------------------------------
LR test of alpha=0: chibar2(01) = 1.0e+04             Prob >= chibar2 = 0.000
```

4a.Outpatient visits - original model

```
. nbreg outpatient i.strokew1 zMoca age i.sex i.edu3 i.emp2 i.medcard i.Disability2 MHcesd_capi,
irr nolog

Negative binomial regression                    Number of obs     =      5,758
                                                LR chi2(10)       =     320.19
Dispersion     = mean                           Prob > chi2       =     0.0000
Log likelihood = -8323.6887                     Pseudo R2         =     0.0189

------------------------------------------------------------------------------------
           outpatient |        IRR   Std. Err.      z    P>|z|     [95% Conf. Interval]
----------------------+-------------------------------------------------------------
             strokew1 |
              stroke  |   1.491691   .2666236     2.24   0.025     1.050838    2.117493
                zMoca |   .9233992   .0251754    -2.92   0.003     .8753515    .9740841
                  age |   .9991229   .0033752    -0.26   0.795     .9925296     1.00576
                      |
                  sex |
              Female  |    .970144   .0461093    -0.64   0.524      .883853     1.06486
                      |
                 edu3 |
       secondary edu  |   .9581131   .0588409    -0.70   0.486     .8494575    1.080667
     third level edu  |   1.048307    .072027     0.69   0.492     .9162296    1.199424
                      |
                 emp2 |
            Employed  |   .6883726   .0408307    -6.30   0.000     .6128225    .7732367
                      |
              medcard |
medcard/gp visit card |   1.308491   .0735854     4.78   0.000      1.17193    1.460964
                      |
          Disability2 |
          Disability  |   1.786977     .12974     8.00   0.000     1.549955    2.060244
          MHcesd_capi |   1.021031   .0035114     6.05   0.000     1.014172    1.027936
                _cons |   1.027715   .2380878     0.12   0.906     .6526458    1.618334
----------------------+-------------------------------------------------------------
             /lnalpha |   .7918733   .0349612                      .7233506     .860396
----------------------+-------------------------------------------------------------
                alpha |   2.207528   .0771779                      2.061328    2.364097
------------------------------------------------------------------------------------
LR test of alpha=0: chibar2(01) = 5125.52             Prob >= chibar2 = 0.000
```

4b.Outpatient visits - model including insurance

```
. nbreg outpatient i.strokew1 zMoca i.ins2 age i.sex i.edu3 i.emp2 i.medcard i.Disability2
MHcesd_capi, irr nolog

Negative binomial regression                    Number of obs     =      5,754
                                                LR chi2(11)       =     320.17
Dispersion     = mean                           Prob > chi2       =     0.0000
Log likelihood = -8316.7611                     Pseudo R2         =     0.0189

------------------------------------------------------------------------------------
           outpatient |        IRR   Std. Err.      z    P>|z|     [95% Conf. Interval]
----------------------+-------------------------------------------------------------
             strokew1 |
              stroke  |   1.490039   .2664271     2.23   0.026     1.049538    2.115423
                zMoca |   .9235939   .0252291    -2.91   0.004     .8754462    .9743896
                      |
                 ins2 |
    private insurance |   1.019028   .0600746     0.32   0.749     .9078318    1.143844
                  age |   .9988237   .0034612    -0.34   0.734     .9920628    1.005631
                      |
                  sex |
              Female  |   .9700035    .046147    -0.64   0.522     .8836458    1.064801
                      |
                 edu3 |
        secondary edu |   .9522039   .0600993    -0.78   0.438     .8414058    1.077592
      third level edu |   1.041242   .0747811     0.56   0.574     .9045215    1.198627
                      |
                 emp2 |
             Employed |   .6871922   .0407845    -6.32   0.000     .6117301    .7719633
                      |
              medcard |
medcard/gp visit card |   1.319573   .0815822     4.49   0.000     1.168983    1.489562
                      |
          Disability2 |
           Disability |   1.790241   .1302135     8.01   0.000     1.552384    2.064542
          MHcesd_capi |    1.02106   .0035179     6.05   0.000     1.014188    1.027978
                _cons |   1.036077   .2404284     0.15   0.879     .6574536    1.632747
----------------------+-------------------------------------------------------------
             /lnalpha |   .7922529   .0349706                       .7237118    .8607939
----------------------+-------------------------------------------------------------
                alpha |   2.208366   .0772278                       2.062073    2.365038
------------------------------------------------------------------------------------
LR test of alpha=0: chibar2(01) = 5124.90             Prob >= chibar2 = 0.000
```

5a.Rehab services used - 3 services (new variable: OT, Psychology and Physiotherapy)

```
. logit SSused3 i.strokew1 zMoca age i.sex i.edu3 i.emp2 i.medcard i.Disability2 MHcesd_capi, or
nolog

Logistic regression                             Number of obs     =      5,760
                                                LR chi2(10)       =     260.77
                                                Prob > chi2       =     0.0000
Log likelihood = -1259.2078                     Pseudo R2         =     0.0938

------------------------------------------------------------------------------------
               SSused3 | Odds Ratio   Std. Err.      z    P>|z|     [95% Conf. Interval]
-----------------------+------------------------------------------------------------
              strokew1 |
               stroke  |   1.247889   .4231788     0.65   0.514      .641978     2.42567
                 zMoca |   .9854331   .0581176    -0.25   0.804     .8778617    1.106186
                   age |   .9876777   .0072565    -1.69   0.091     .9735571    1.002003
                       |
                   sex |
               Female  |   1.139399    .129593     1.15   0.251     .9117204    1.423934
                       |
                  edu3 |
        secondary edu  |   1.447394   .2076175     2.58   0.010     1.092668    1.917278
      third level edu  |   1.763759   .2863017     3.50   0.000     1.283122    2.424433
                       |
                  emp2 |
             Employed  |   .6002524   .0949352    -3.23   0.001     .4402596    .8183875
                       |
               medcard |
medcard/gp visit card  |   2.697211   .3800268     7.04   0.000     2.046369    3.555053
                       |
           Disability2 |
           Disability  |   2.593357   .3499073     7.06   0.000     1.990739    3.378394
           MHcesd_capi |   1.039322   .0066217     6.05   0.000     1.026425    1.052382
                 _cons |   .0424572   .0217686    -6.16   0.000     .0155426    .1159789
------------------------------------------------------------------------------------
```

5b. Rehab services used (3 services) - model including insurance

```
. logit SSused3 i.strokew1 zMoca i.ins2 age i.sex i.edu3 i.emp2 i.medcard i.Disability2
MHcesd_capi, or nolog

Logistic regression                              Number of obs     =      5,756
                                                 LR chi2(11)       =     256.37
                                                 Prob > chi2       =     0.0000
Log likelihood = -1258.4763                      Pseudo R2         =     0.0924

------------------------------------------------------------------------------------------
               SSused3 | Odds Ratio   Std. Err.      z    P>|z|     [95% Conf. Interval]
----------------------+-------------------------------------------------------------------
              strokew1 |
               stroke  |   1.255797   .4256175     0.67   0.502     .6462916    2.440117
                 zMoca |   .9838256   .0582233    -0.28   0.783     .8760797    1.104823
                       |
                  ins2 |
    private insurance  |   .9642815   .1269263    -0.28   0.782     .7450098    1.248089
                   age |   .9883855   .0075732    -1.52   0.127     .9736531    1.003341
                       |
                   sex |
               Female  |   1.138567   .1295439     1.14   0.254     .9109836    1.423006
                       |
                  edu3 |
        secondary edu  |   1.451313   .2120397     2.55   0.011     1.089931    1.932517
      third level edu  |   1.779988   .2984687     3.44   0.001     1.281408    2.472559
                       |
                  emp2 |
             Employed  |   .6015445   .0951988    -3.21   0.001     .4411222    .8203074
                       |
               medcard |
medcard/gp visit card  |   2.651081    .401044     6.44   0.000     1.970866    3.566064
                       |
           Disability2 |
           Disability  |    2.57538   .3492746     6.98   0.000     1.974244    3.359555
           MHcesd_capi |   1.038788   .0066504     5.94   0.000     1.025835    1.051905
                 _cons |   .0418651   .0215945    -6.15   0.000     .0152333    .1150564
------------------------------------------------------------------------------------------
```

6a.Rehab services used - 2 services only (original variable: OT and Psychology)

```
. logit RehabUsed2 i.strokew1 zMoca age i.sex i.edu3 i.emp2 i.medcard i.Disability2 MHcesd_capi,
or nolog

Logistic regression                             Number of obs     =      5,760
                                                LR chi2(10)       =     160.85
                                                Prob > chi2       =     0.0000
Log likelihood = -533.39536                     Pseudo R2         =     0.1310

--------------------------------------------------------------------------------------
           RehabUsed2 | Odds Ratio   Std. Err.      z    P>|z|     [95% Conf. Interval]
----------------------+---------------------------------------------------------------
             strokew1 |
              stroke  |   3.023982   1.210921     2.76   0.006      1.37951    6.628779
                zMoca |   .9380939   .0918678    -0.65   0.514     .7742619    1.136592
                  age |   .9660501   .0117159    -2.85   0.004     .9433582    .9892879
                      |
                  sex |
              Female  |     .98947   .1873641    -0.06   0.955     .6826857    1.434117
                      |
                 edu3 |
       secondary edu  |   1.573118   .3827416     1.86   0.063     .9764793     2.53431
     third level edu  |   2.043387   .5582288     2.62   0.009     1.196224    3.490509
                      |
                 emp2 |
            Employed  |   .4544699   .1282731    -2.79   0.005     .2613698    .7902326
                      |
              medcard |
medcard/gp visit card  |   2.865971   .6841755     4.41   0.000     1.795022    4.575872
                      |
          Disability2 |
          Disability  |   3.386041   .7127287     5.79   0.000     2.241416    5.115191
          MHcesd_capi |   1.048094   .0098247     5.01   0.000     1.029013    1.067528
                _cons |   .0442429   .0373463    -3.69   0.000     .0084591    .2313985
--------------------------------------------------------------------------------------
```

6b. Rehab services used (2 services) - model including insurance

```
. logit RehabUsed2 i.strokew1 zMoca i.ins2 age i.sex i.edu3 i.emp2 i.medcard i.Disability2
MHcesd_capi, or nolog

Logistic regression                             Number of obs     =      5,756
                                                LR chi2(11)       =     155.13
                                                Prob > chi2       =     0.0000
Log likelihood = -532.37525                     Pseudo R2         =     0.1272

------------------------------------------------------------------------------------
           RehabUsed2 | Odds Ratio   Std. Err.      z    P>|z|     [95% Conf. Interval]
----------------------+-------------------------------------------------------------
             strokew1 |
              stroke  |   3.065494   1.226442     2.80   0.005     1.399433    6.715045
                zMoca |   .9359527   .0920593    -0.67   0.501      .771846    1.134951
                      |
                 ins2 |
    private insurance |   .9612339   .2131456    -0.18   0.858     .6224182    1.484485
                  age |   .9669326   .0123155    -2.64   0.008     .9430935    .9913744
                      |
                  sex |
              Female  |   .9854501   .1869269    -0.08   0.938     .6794741    1.429211
                      |
                 edu3 |
        secondary edu |   1.558739   .3853763     1.80   0.073     .9601209    2.530586
      third level edu |   2.051816   .5775512     2.55   0.011     1.181788    3.562354
                      |
                 emp2 |
            Employed  |   .4557496   .1288219    -2.78   0.005     .2618945    .7930971
                      |
              medcard |
medcard/gp visit card |   2.804016   .7223069     4.00   0.000      1.69244    4.645662
                      |
          Disability2 |
           Disability |   3.346762   .7095832     5.70   0.000      2.20878    5.071043
          MHcesd_capi |   1.046822   .0099186     4.83   0.000     1.027561    1.066444
                _cons |   .0439556   .0374328    -3.67   0.000     .0082819    .2332924
------------------------------------------------------------------------------------

*******************************************************************************************
```

3.Multiple regressions including *cover* (0=no cover;1=medical card or GP card only/ 2=private insurance only/ 3=dual cover) as a covariate instead of *medcard*

```
. tab cover

            cover |      Freq.     Percent        Cum.
------------------+-----------------------------------
         No cover |        842       10.32       10.32
Medical card only |      2,621       32.11       42.43
   Insurance only |      3,281       40.20       82.63
       Dual cover |      1,418       17.37      100.00
------------------+-----------------------------------
            Total |      8,162      100.00
```

1a.GP visits - original model

```
. nbreg gpvisits i.strokew1 zMoca age i.sex i.edu3 i.emp2 i.medcard i.Disability2 MHcesd_capi,
irr nolog

Negative binomial regression                    Number of obs     =      5,753
                                                LR chi2(10)       =    1364.13
Dispersion     = mean                           Prob > chi2       =     0.0000
Log likelihood = -13146.315                     Pseudo R2         =     0.0493

--------------------------------------------------------------------------------------
            gpvisits |        IRR   Std. Err.      z    P>|z|     [95% Conf. Interval]
---------------------+----------------------------------------------------------------
            strokew1 |
              stroke  |    1.26931   .1088749     2.78   0.005     1.072892    1.501687
               zMoca |   1.066888   .0136849     5.05   0.000     1.040401     1.09405
                 age |   1.003391   .0016149     2.10   0.035     1.000231    1.006561
                     |
                 sex |
              Female  |   1.016083   .0235785     0.69   0.492     .9709047    1.063363
                     |
                edu3 |
       secondary edu  |   .9953264   .0292704    -0.16   0.873     .9395795    1.054381
     third level edu  |   .9537152   .0318513    -1.42   0.156     .8932871    1.018231
                     |
                emp2 |
            Employed  |   .8489824   .0245915    -5.65   0.000     .8021266    .8985752
                     |
             medcard |
medcard/gp visit card  |   1.597204   .0444586    16.82   0.000     1.512401    1.686762
                     |
         Disability2 |
```

```
           Disability  |   1.434818    .050219    10.32   0.000     1.339691      1.5367
           MHcesd_capi |   1.018779   .0016563    11.44   0.000     1.015538     1.02203
                 _cons |   2.029435    .225706     6.36   0.000     1.631953    2.523728
-----------------------+----------------------------------------------------------------
              /lnalpha |  -.8281074   .0318193                     -.8904721   -.7657428
-----------------------+----------------------------------------------------------------
                 alpha |   .4368753   .0139011                      .4104619    .4649884
----------------------------------------------------------------------------------------
LR test of alpha=0: chibar2(01) = 4075.71             Prob >= chibar2 = 0.000
```

1b.GP visits - model including cover instead of medical card

```
. nbreg gpvisits i.strokew1 zMoca age i.sex i.edu3 i.emp2 i.cover i.Disability2 MHcesd_capi, irr
nolog

Negative binomial regression                    Number of obs     =      5,749
                                                LR chi2(12)       =    1371.17
Dispersion     = mean                           Prob > chi2       =     0.0000
Log likelihood = -13128.512                     Pseudo R2         =     0.0496

------------------------------------------------------------------------------------
          gpvisits |        IRR   Std. Err.      z    P>|z|     [95% Conf. Interval]
-------------------+----------------------------------------------------------------
          strokew1 |
           stroke  |   1.264975    .108289     2.75   0.006     1.069582    1.496062
             zMoca |   1.065209   .0137042     4.91   0.000     1.038685     1.09241
               age |   1.004104   .0016661     2.47   0.014     1.000844    1.007375
                   |
               sex |
           Female  |   1.013742   .0235072     0.59   0.556     .9686999    1.060878
                   |
              edu3 |
    secondary edu  |   1.003761   .0301187     0.13   0.900     .9464321    1.064563
  third level edu  |   .9558508   .0327583    -1.32   0.188     .8937547    1.022261
                   |
              emp2 |
         Employed  |   .8546409   .0247762    -5.42   0.000     .8074344    .9046074
                   |
             cover |
Medical card only  |    1.85272   .0863508    13.23   0.000     1.690976    2.029936
   Insurance only  |   1.148018   .0497806     3.18   0.001      1.05448    1.249852
       Dual cover  |   1.692786   .0849942    10.48   0.000     1.534135    1.867844
                   |
       Disability2 |
       Disability  |   1.428096   .0499838    10.18   0.000     1.333415    1.529501
       MHcesd_capi |   1.018661   .0016601    11.34   0.000     1.015412     1.02192
             _cons |   1.725882   .2030493     4.64   0.000     1.370464    2.173474
-------------------+----------------------------------------------------------------
          /lnalpha |  -.8331272    .031896                     -.8956423   -.7706122
-------------------+----------------------------------------------------------------
             alpha |   .4346878   .0138648                      .4083452    .4627297
------------------------------------------------------------------------------------
LR test of alpha=0: chibar2(01) = 4045.44             Prob >= chibar2 = 0.000
```

2a.Emergency visits - original model

```
. nbreg emergency i.strokew1 zMoca age i.sex i.edu3 i.emp2 i.medcard i.Disability2 MHcesd_capi,
irr nolog

Negative binomial regression                    Number of obs     =      5,757
                                                LR chi2(10)       =     136.19
Dispersion     = mean                           Prob > chi2       =     0.0000
Log likelihood = -3156.5347                     Pseudo R2         =     0.0211

------------------------------------------------------------------------------------------
             emergency |        IRR   Std. Err.      z    P>|z|     [95% Conf. Interval]
-----------------------+------------------------------------------------------------------
              strokew1 |
               stroke  |   1.562992   .4085484     1.71   0.088      .936402    2.608863
                 zMoca |   1.055994   .0455497     1.26   0.207     .9703874    1.149152
                   age |   1.000074   .0053157     0.01   0.989     .9897096    1.010547
                       |
                   sex |
               Female  |   .9694865   .0738441    -0.41   0.684       .83504     1.12558
                       |
                  edu3 |
        secondary edu  |    .981302   .0952215    -0.19   0.846      .811345    1.186861
      third level edu  |   1.150641   .1256091     1.29   0.199     .9290065     1.42515
                       |
                  emp2 |
             Employed  |   .9343425   .0892415    -0.71   0.477     .7748286    1.126696
                       |
               medcard |
medcard/gp visit card  |   1.425844   .1318271     3.84   0.000     1.189525    1.709111
                       |
           Disability2 |
           Disability  |   1.880549   .1998814     5.94   0.000     1.526903    2.316103
           MHcesd_capi |   1.021914   .0050527     4.38   0.000     1.012059    1.031865
                 _cons |   .1416426   .0518411    -5.34   0.000     .0691282    .2902237
-----------------------+------------------------------------------------------------------
              /lnalpha |   1.135652    .078928                      .9809563    1.290348
-----------------------+------------------------------------------------------------------
                 alpha |   3.113204   .2457189                      2.667005    3.634052
------------------------------------------------------------------------------------------
LR test of alpha=0: chibar2(01) = 644.50              Prob >= chibar2 = 0.000
```

2b.Emergency visits - model including cover instead of medical card

```
. nbreg emergency i.strokew1 zMoca age i.sex i.edu3 i.emp2 i.cover i.Disability2 MHcesd_capi, irr
nolog

Negative binomial regression                    Number of obs     =      5,753
                                                LR chi2(12)       =     133.02
Dispersion     = mean                           Prob > chi2       =     0.0000
Log likelihood =  -3150.155                     Pseudo R2         =     0.0207

------------------------------------------------------------------------------------
         emergency |        IRR   Std. Err.      z    P>|z|     [95% Conf. Interval]
-------------------+----------------------------------------------------------------
          strokew1 |
           stroke  |   1.565694   .4081133     1.72   0.085     .9393622    2.609642
             zMoca |   1.050141   .0453807     1.13   0.258     .9648586     1.14296
               age |   1.001894   .0054743     0.35   0.729     .9912222    1.012682
                   |
               sex |
           Female  |   .9726593   .0741259    -0.36   0.716      .837705    1.129355
                   |
              edu3 |
    secondary edu  |   1.000985    .099586     0.01   0.992     .8236506    1.216499
  third level edu  |   1.198483   .1351645     1.61   0.108     .9608019    1.494962
                   |
              emp2 |
         Employed  |   .9324084    .089132    -0.73   0.464     .7731024    1.124541
                   |
             cover |
Medical card only  |   1.241408    .182592     1.47   0.142      .930499    1.656201
   Insurance only  |   .8060051    .109816    -1.58   0.113     .6171119    1.052717
       Dual cover  |   1.133766   .1812589     0.79   0.432     .8287793    1.550985
                   |
       Disability2 |
       Disability  |    1.84943   .1969827     5.77   0.000     1.500985    2.278764
       MHcesd_capi |   1.020777   .0050898     4.12   0.000     1.010849    1.030801
             _cons |   .1481228   .0567526    -4.98   0.000     .0699018    .3138744
-------------------+----------------------------------------------------------------
          /lnalpha |   1.131259   .0791762                      .9760763    1.286442
-------------------+----------------------------------------------------------------
             alpha |   3.099556   .2454112                      2.654022    3.619882
------------------------------------------------------------------------------------
LR test of alpha=0: chibar2(01) = 635.39               Prob >= chibar2 = 0.000
```

3a.Nights in hospital - original model

```
. nbreg nights i.strokew1 zMoca age i.sex i.edu3 i.emp2 i.medcard i.Disability2 MHcesd_capi, irr
nolog

Negative binomial regression                    Number of obs     =      5,758
                                                LR chi2(10)       =     100.85
Dispersion     = mean                           Prob > chi2       =     0.0000
Log likelihood = -4043.7539                     Pseudo R2         =     0.0123

------------------------------------------------------------------------------------------
               nights |        IRR   Std. Err.      z    P>|z|     [95% Conf. Interval]
----------------------+-------------------------------------------------------------------
             strokew1 |
              stroke  |   1.930541   .8994416     1.41   0.158     .7746455    4.811217
                zMoca |   1.047743   .0662378     0.74   0.461       .92564    1.185952
                  age |   1.021044   .0088188     2.41   0.016     1.003905    1.038475
                      |
                  sex |
               Female |   .9332921   .1110405    -0.58   0.562     .7391694    1.178396
                      |
                 edu3 |
        secondary edu |   1.233323   .1891828     1.37   0.172     .9130801    1.665885
      third level edu |   1.251192   .2208183     1.27   0.204     .8853166    1.768274
                      |
                 emp2 |
             Employed |   .7813591   .1203808    -1.60   0.109     .5777096    1.056798
                      |
              medcard |
medcard/gp visit card |   1.518647   .2199657     2.88   0.004     1.143315    2.017193
                      |
          Disability2 |
           Disability |   2.106449   .3963532     3.96   0.000     1.456758    3.045892
          MHcesd_capi |     1.0189   .0088877     2.15   0.032     1.001628    1.036469
                _cons |   .0973825   .0587259    -3.86   0.000     .0298655    .3175357
----------------------+-------------------------------------------------------------------
             /lnalpha |   2.852873   .0487549                      2.757315    2.948431
----------------------+-------------------------------------------------------------------
                alpha |   17.33752   .8452886                      15.75748    19.07599
------------------------------------------------------------------------------------------
LR test of alpha=0: chibar2(01) = 1.0e+04              Prob >= chibar2 = 0.000
```

3b.Nights in hospital - model including cover instead of medical card

```
. nbreg nights i.strokew1 zMoca age i.sex i.edu3 i.emp2 i.cover i.Disability2 MHcesd_capi, irr
nolog

Negative binomial regression                    Number of obs     =      5,754
                                                LR chi2(12)       =     105.42
Dispersion     = mean                           Prob > chi2       =     0.0000
Log likelihood = -4037.8908                     Pseudo R2         =     0.0129

------------------------------------------------------------------------------------
           nights |        IRR   Std. Err.      z    P>|z|     [95% Conf. Interval]
------------------+-----------------------------------------------------------------
         strokew1 |
          stroke  |   1.892449   .8805335     1.37   0.170     .7602742    4.710618
            zMoca |   1.042986   .0660888     0.66   0.507     .9211749    1.180905
              age |   1.021487   .0089723     2.42   0.016     1.004053    1.039225
                  |
              sex |
          Female  |   .9059521   .1088196    -0.82   0.411      .715916    1.146432
                  |
             edu3 |
    secondary edu |   1.220133   .1933312     1.26   0.209     .8944033    1.664489
  third level edu |   1.222751   .2235265     1.10   0.271     .8545412    1.749617
                  |
             emp2 |
         Employed |   .8079196   .1252901    -1.38   0.169     .5961643     1.09489
                  |
            cover |
Medical card only |   2.359292   .5711878     3.55   0.000     1.467927    3.791916
   Insurance only |   1.544237   .3373096     1.99   0.047     1.006433    2.369425
       Dual cover |    1.99021   .5185509     2.64   0.008     1.194312    3.316502
                  |
      Disability2 |
       Disability |    2.13453    .402248     4.02   0.000      1.47535    3.088229
      MHcesd_capi |   1.019101   .0089224     2.16   0.031     1.001763     1.03674
            _cons |   .0663251   .0413687    -4.35   0.000     .0195327    .2252133
------------------+-----------------------------------------------------------------
         /lnalpha |   2.849256   .0488281                      2.753554    2.944957
------------------+-----------------------------------------------------------------
            alpha |   17.27492   .8435024                      15.69833    19.00985
------------------------------------------------------------------------------------
LR test of alpha=0: chibar2(01) = 1.0e+04             Prob >= chibar2 = 0.000
```

4a.Outpatient visits - original model

```
. nbreg outpatient i.strokew1 zMoca age i.sex i.edu3 i.emp2 i.medcard i.Disability2 MHcesd_capi,
irr nolog

Negative binomial regression                    Number of obs     =      5,758
                                                LR chi2(10)       =     320.19
Dispersion     = mean                           Prob > chi2       =     0.0000
Log likelihood = -8323.6887                     Pseudo R2         =     0.0189

------------------------------------------------------------------------------------------
            outpatient |        IRR   Std. Err.      z    P>|z|     [95% Conf. Interval]
-----------------------+------------------------------------------------------------------
              strokew1 |
               stroke  |   1.491691   .2666236     2.24   0.025     1.050838    2.117493
                 zMoca |   .9233992   .0251754    -2.92   0.003     .8753515    .9740841
                   age |   .9991229   .0033752    -0.26   0.795     .9925296     1.00576
                       |
                   sex |
               Female  |    .970144   .0461093    -0.64   0.524      .883853     1.06486
                       |
                  edu3 |
        secondary edu  |   .9581131   .0588409    -0.70   0.486     .8494575    1.080667
      third level edu  |   1.048307    .072027     0.69   0.492     .9162296    1.199424
                       |
                  emp2 |
             Employed  |   .6883726   .0408307    -6.30   0.000     .6128225    .7732367
                       |
               medcard |
medcard/gp visit card  |   1.308491   .0735854     4.78   0.000      1.17193    1.460964
                       |
           Disability2 |
           Disability  |   1.786977     .12974     8.00   0.000     1.549955    2.060244
           MHcesd_capi |   1.021031   .0035114     6.05   0.000     1.014172    1.027936
                 _cons |   1.027715   .2380878     0.12   0.906     .6526458    1.618334
-----------------------+------------------------------------------------------------------
              /lnalpha |   .7918733   .0349612                      .7233506     .860396
-----------------------+------------------------------------------------------------------
                 alpha |   2.207528   .0771779                      2.061328    2.364097
------------------------------------------------------------------------------------------
LR test of alpha=0: chibar2(01) = 5125.52             Prob >= chibar2 = 0.000
```

4b.Outpatient visits - model including cover instead of medical card

```
. nbreg outpatient i.strokew1 zMoca age i.sex i.edu3 i.emp2 i.cover i.Disability2 MHcesd_capi,
irr nolog

Negative binomial regression                    Number of obs     =      5,754
                                                LR chi2(12)       =     328.39
Dispersion     = mean                           Prob > chi2       =     0.0000
Log likelihood = -8312.6511                     Pseudo R2         =     0.0194

------------------------------------------------------------------------------------
        outpatient |        IRR   Std. Err.      z    P>|z|     [95% Conf. Interval]
-------------------+----------------------------------------------------------------
          strokew1 |
           stroke  |   1.486677   .2653898     2.22   0.026     1.047772    2.109436
             zMoca |   .9232165   .0252165    -2.92   0.003     .8750928    .9739866
               age |    .999673   .0034779    -0.09   0.925     .9928795    1.006513
                   |
               sex |
           Female  |   .9633728   .0458543    -0.78   0.433     .8775647    1.057571
                   |
              edu3 |
    secondary edu  |    .962892   .0608211    -0.60   0.549     .8507685    1.089792
  third level edu  |   1.043898   .0748138     0.60   0.549     .9070987    1.201329
                   |
              emp2 |
         Employed  |   .6971448   .0414856    -6.06   0.000     .6203972    .7833867
                   |
             cover |
Medical card only  |   1.629241   .1552309     5.12   0.000     1.351713     1.96375
   Insurance only  |   1.234193   .1088806     2.39   0.017     1.038222    1.467156
       Dual cover  |   1.460638   .1515774     3.65   0.000     1.191816    1.790094
                   |
       Disability2 |
       Disability  |   1.791215   .1301236     8.02   0.000     1.553502    2.065302
       MHcesd_capi |   1.020873   .0035149     6.00   0.000     1.014007    1.027785
             _cons |   .8320982   .2029429    -0.75   0.451     .5159083    1.342075
-------------------+----------------------------------------------------------------
          /lnalpha |   .7884059   .0350221                       .7197639    .8570479
-------------------+----------------------------------------------------------------
             alpha |   2.199887   .0770446                       2.053948    2.356195
------------------------------------------------------------------------------------
LR test of alpha=0: chibar2(01) = 5101.29             Prob >= chibar2 = 0.000
```

5a.Rehab services used - 3 services (new variable: OT, Psychology and Physiotherapy)

```
. logit SSused3 i.strokew1 zMoca age i.sex i.edu3 i.emp2 i.medcard i.Disability2 MHcesd_capi, or
nolog

Logistic regression                             Number of obs     =      5,760
                                                LR chi2(10)       =     260.77
                                                Prob > chi2       =     0.0000
Log likelihood = -1259.2078                     Pseudo R2         =     0.0938

------------------------------------------------------------------------------------
              SSused3 | Odds Ratio   Std. Err.      z    P>|z|     [95% Conf. Interval]
----------------------+-------------------------------------------------------------
             strokew1 |
               stroke  |   1.247889   .4231788     0.65   0.514      .641978     2.42567
                zMoca |   .9854331   .0581176    -0.25   0.804     .8778617    1.106186
                  age |   .9876777   .0072565    -1.69   0.091     .9735571    1.002003
                      |
                  sex |
               Female  |   1.139399    .129593     1.15   0.251     .9117204    1.423934
                      |
                 edu3 |
        secondary edu  |   1.447394   .2076175     2.58   0.010     1.092668    1.917278
      third level edu  |   1.763759   .2863017     3.50   0.000     1.283122    2.424433
                      |
                 emp2 |
             Employed  |   .6002524   .0949352    -3.23   0.001     .4402596    .8183875
                      |
              medcard |
medcard/gp visit card  |   2.697211   .3800268     7.04   0.000     2.046369    3.555053
                      |
          Disability2 |
           Disability  |   2.593357   .3499073     7.06   0.000     1.990739    3.378394
          MHcesd_capi |   1.039322   .0066217     6.05   0.000     1.026425    1.052382
                _cons |   .0424572   .0217686    -6.16   0.000     .0155426    .1159789
------------------------------------------------------------------------------------
```

5b. Rehab services used (3 services) - model including cover instead of medical card

```
. logit SSused3 i.strokew1 zMoca age i.sex i.edu3 i.emp2 i.cover i.Disability2 MHcesd_capi, or
nolog

Logistic regression                             Number of obs     =      5,756
                                                LR chi2(12)       =     256.55
                                                Prob > chi2       =     0.0000
Log likelihood = -1258.3851                     Pseudo R2         =     0.0925

------------------------------------------------------------------------------------
           SSused3 | Odds Ratio   Std. Err.      z    P>|z|     [95% Conf. Interval]
-------------------+----------------------------------------------------------------
          strokew1 |
           stroke  |   1.256403   .4257708     0.67   0.501     .6466561    2.441096
             zMoca |   .9833193   .0581988    -0.28   0.776     .8756193    1.104266
               age |   .9886388   .0075956    -1.49   0.137     .9738632    1.003639
                   |
               sex |
           Female  |   1.137502    .129451     1.13   0.258     .9100875    1.421744
                   |
              edu3 |
    secondary edu  |   1.456617   .2133298     2.57   0.010     1.093155    1.940924
  third level edu  |   1.781957   .2991283     3.44   0.001      1.28236    2.476191
                   |
              emp2 |
         Employed  |   .6031985   .0955378    -3.19   0.001     .4422241    .8227693
                   |
             cover |
Medical card only  |   2.899676   .7599295     4.06   0.000     1.734889    4.846489
   Insurance only  |   1.061058   .2789838     0.23   0.822     .6337714    1.776419
       Dual cover  |   2.715398   .7542479     3.60   0.000     1.575425    4.680251
                   |
       Disability2 |
       Disability  |   2.572788   .3490275     6.97   0.000       1.9721    3.356441
       MHcesd_capi |   1.038826   .0066516     5.95   0.000     1.025871    1.051945
             _cons |   .0380032   .0214693    -5.79   0.000     .0125588    .1149985
------------------------------------------------------------------------------------
```

6a.Rehab services used- 2 services only (original variable: OT and Psychology)

```
. logit RehabUsed2 i.strokew1 zMoca age i.sex i.edu3 i.emp2 i.medcard i.Disability2 MHcesd_capi,
or nolog

Logistic regression                             Number of obs     =      5,760
                                                LR chi2(10)       =     160.85
                                                Prob > chi2       =     0.0000
Log likelihood = -533.39536                     Pseudo R2         =     0.1310

------------------------------------------------------------------------------------
           RehabUsed2 | Odds Ratio   Std. Err.      z    P>|z|     [95% Conf. Interval]
----------------------+-------------------------------------------------------------
             strokew1 |
              stroke  |   3.023982   1.210921     2.76   0.006      1.37951    6.628779
                zMoca |   .9380939   .0918678    -0.65   0.514     .7742619    1.136592
                  age |   .9660501   .0117159    -2.85   0.004     .9433582    .9892879
                      |
                  sex |
              Female  |     .98947   .1873641    -0.06   0.955     .6826857    1.434117
                      |
                 edu3 |
       secondary edu  |   1.573118   .3827416     1.86   0.063     .9764793     2.53431
     third level edu  |   2.043387   .5582288     2.62   0.009     1.196224    3.490509
                      |
                 emp2 |
            Employed  |   .4544699   .1282731    -2.79   0.005     .2613698    .7902326
                      |
              medcard |
medcard/gp visit card |   2.865971   .6841755     4.41   0.000     1.795022    4.575872
                      |
          Disability2 |
          Disability  |   3.386041   .7127287     5.79   0.000     2.241416    5.115191
          MHcesd_capi |   1.048094   .0098247     5.01   0.000     1.029013    1.067528
                _cons |   .0442429   .0373463    -3.69   0.000     .0084591    .2313985
------------------------------------------------------------------------------------
```

6b.Rehab services used (2 services) - model including cover instead of medical card

```
. logit RehabUsed2 i.strokew1 zMoca age i.sex i.edu3 i.emp2 i.cover i.Disability2 MHcesd_capi, or
nolog

Logistic regression                             Number of obs     =      5,756
                                                LR chi2(12)       =     155.91
                                                Prob > chi2       =     0.0000
Log likelihood = -531.98916                     Pseudo R2         =     0.1278

------------------------------------------------------------------------------------
        RehabUsed2 | Odds Ratio   Std. Err.      z    P>|z|     [95% Conf. Interval]
-------------------+----------------------------------------------------------------
          strokew1 |
           stroke  |   3.073338   1.229636     2.81   0.005     1.402963    6.732468
             zMoca |   .9343493   .0918771    -0.69   0.490     .7705632    1.132949
               age |   .9677374   .0123483    -2.57   0.010     .9438352    .9922449
                   |
               sex |
           Female  |   .9839554   .1866755    -0.09   0.932     .6784002    1.427134
                   |
              edu3 |
    secondary edu  |   1.574888   .3905416     1.83   0.067     .9686534    2.560538
  third level edu  |   2.057317   .5810903     2.55   0.011     1.182711    3.578685
                   |
              emp2 |
         Employed  |    .458917   .1297986    -2.75   0.006     .2636228    .7988868
                   |
             cover |
Medical card only  |   3.923297   1.912195     2.80   0.005     1.509315    10.19818
   Insurance only  |    1.38805   .6902024     0.66   0.510     .5237797    3.678423
       Dual cover  |   3.428856   1.772495     2.38   0.017     1.244898    9.444193
                   |
       Disability2 |
       Disability  |   3.348344   .7102538     5.70   0.000     2.209391    5.074436
       MHcesd_capi |   1.046956   .0099264     4.84   0.000      1.02768    1.066593
             _cons |   .0305717   .0293373    -3.63   0.000     .0046611    .2005168
------------------------------------------------------------------------------------
```

```
*************************************************************************************
```

4. Multiple regressions including long-term illness (*longterm*) as a covariate; attempt to measure other non-CVD conditions which could impact HSU

```
. tab longterm

           longterm |      Freq.     Percent        Cum.
--------------------+-----------------------------------
         No illness |      5,039       61.68       61.68
Yes longterm illness |      3,131       38.32      100.00
--------------------+-----------------------------------
              Total |      8,170      100.00
```

1a.GP visits - original model

```
. nbreg gpvisits i.strokew1 zMoca age i.sex i.edu3 i.emp2 i.medcard i.Disability2 MHcesd_capi,
irr nolog

Negative binomial regression                    Number of obs     =      5,753
                                                LR chi2(10)       =    1364.13
Dispersion     = mean                           Prob > chi2       =     0.0000
Log likelihood = -13146.315                     Pseudo R2         =     0.0493

---------------------------------------------------------------------------------------
             gpvisits |        IRR   Std. Err.      z    P>|z|     [95% Conf. Interval]
----------------------+----------------------------------------------------------------
             strokew1 |
              stroke  |    1.26931   .1088749     2.78   0.005     1.072892    1.501687
                zMoca |   1.066888   .0136849     5.05   0.000     1.040401     1.09405
                  age |   1.003391   .0016149     2.10   0.035     1.000231    1.006561
                      |
                  sex |
              Female  |   1.016083   .0235785     0.69   0.492     .9709047    1.063363
                      |
                 edu3 |
       secondary edu  |   .9953264   .0292704    -0.16   0.873     .9395795    1.054381
     third level edu  |   .9537152   .0318513    -1.42   0.156     .8932871    1.018231
                      |
                 emp2 |
            Employed  |   .8489824   .0245915    -5.65   0.000     .8021266    .8985752
                      |
              medcard |
medcard/gp visit card |   1.597204   .0444586    16.82   0.000     1.512401    1.686762
                      |
          Disability2 |
          Disability  |   1.434818    .050219    10.32   0.000     1.339691      1.5367
          MHcesd_capi |   1.018779   .0016563    11.44   0.000     1.015538     1.02203
```

```
                 _cons |   2.029435    .225706     6.36   0.000     1.631953    2.523728
-----------------------+----------------------------------------------------------------
              /lnalpha |  -.8281074   .0318193                     -.8904721   -.7657428
-----------------------+----------------------------------------------------------------
                 alpha |   .4368753   .0139011                      .4104619    .4649884
----------------------------------------------------------------------------------------
LR test of alpha=0: chibar2(01) = 4075.71             Prob >= chibar2 = 0.000
```

1b.GP visits - model including longterm illness

```
. nbreg gpvisits i.strokew1 zMoca i.longterm age i.sex i.edu3 i.emp2 i.medcard i.Disability2
MHcesd_capi, irr nolog

Negative binomial regression                    Number of obs     =      5,748
                                                LR chi2(11)       =    1618.78
Dispersion     = mean                           Prob > chi2       =     0.0000
Log likelihood = -13007.973                     Pseudo R2         =     0.0586

------------------------------------------------------------------------------------
            gpvisits |        IRR   Std. Err.      z    P>|z|     [95% Conf. Interval]
---------------------+--------------------------------------------------------------
            strokew1 |
             stroke  |   1.203119   .1004988     2.21   0.027     1.021424    1.417134
               zMoca |   1.072254   .0134819     5.55   0.000     1.046153    1.099006
                     |
            longterm |
 Yes longterm illness  |   1.470992   .0351196    16.17   0.000     1.403744    1.541461
                 age |   1.004484   .0015881     2.83   0.005     1.001376    1.007602
                     |
                 sex |
             Female  |   1.011344   .0230472     0.49   0.621     .9671661     1.05754
                     |
                edu3 |
       secondary edu  |   .9970944    .028727    -0.10   0.920     .9423506    1.055018
     third level edu  |   .9426187   .0308805    -1.80   0.071     .8839962    1.005129
                     |
                emp2 |
           Employed  |   .8892008   .0254868    -4.10   0.000     .8406248    .9405838
                     |
             medcard |
medcard/gp visit card  |   1.544167   .0423642    15.84   0.000     1.463328    1.629472
                     |
         Disability2 |
         Disability  |   1.266007   .0443783     6.73   0.000     1.181948    1.356045
         MHcesd_capi |   1.015355   .0016221     9.54   0.000     1.012181    1.018539
               _cons |   1.664557   .1829528     4.64   0.000     1.341969     2.06469
---------------------+--------------------------------------------------------------
            /lnalpha |  -.9043947   .0327966                      -.9686748   -.8401145
---------------------+--------------------------------------------------------------
               alpha |   .4047868   .0132756                       .3795857    .4316611
------------------------------------------------------------------------------------
LR test of alpha=0: chibar2(01) = 3700.88             Prob >= chibar2 = 0.000
```

2a.Emergency visits - original model

```
. nbreg emergency i.strokew1 zMoca age i.sex i.edu3 i.emp2 i.medcard i.Disability2 MHcesd_capi,
irr nolog

Negative binomial regression                    Number of obs     =      5,757
                                                LR chi2(10)       =     136.19
Dispersion     = mean                           Prob > chi2       =     0.0000
Log likelihood = -3156.5347                     Pseudo R2         =     0.0211

------------------------------------------------------------------------------------------
             emergency |        IRR   Std. Err.      z    P>|z|     [95% Conf. Interval]
-----------------------+------------------------------------------------------------------
              strokew1 |
               stroke  |   1.562992   .4085484     1.71   0.088      .936402    2.608863
                 zMoca |   1.055994   .0455497     1.26   0.207     .9703874    1.149152
                   age |   1.000074   .0053157     0.01   0.989     .9897096    1.010547
                       |
                   sex |
               Female  |   .9694865   .0738441    -0.41   0.684       .83504     1.12558
                       |
                  edu3 |
        secondary edu  |    .981302   .0952215    -0.19   0.846      .811345    1.186861
      third level edu  |   1.150641   .1256091     1.29   0.199     .9290065     1.42515
                       |
                  emp2 |
             Employed  |   .9343425   .0892415    -0.71   0.477     .7748286    1.126696
                       |
               medcard |
medcard/gp visit card  |   1.425844   .1318271     3.84   0.000     1.189525    1.709111
                       |
           Disability2 |
           Disability  |   1.880549   .1998814     5.94   0.000     1.526903    2.316103
           MHcesd_capi |   1.021914   .0050527     4.38   0.000     1.012059    1.031865
                 _cons |   .1416426   .0518411    -5.34   0.000     .0691282    .2902237
-----------------------+------------------------------------------------------------------
              /lnalpha |   1.135652    .078928                      .9809563    1.290348
-----------------------+------------------------------------------------------------------
                 alpha |   3.113204   .2457189                      2.667005    3.634052
------------------------------------------------------------------------------------------
LR test of alpha=0: chibar2(01) = 644.50              Prob >= chibar2 = 0.000
```

2b.Emergency visits - model including longterm illness

```
. nbreg emergency i.strokew1 zMoca i.longterm age i.sex i.edu3 i.emp2 i.medcard i.Disability2
MHcesd_capi, irr nolog

Negative binomial regression                    Number of obs     =      5,752
                                                LR chi2(11)       =     192.59
Dispersion     = mean                           Prob > chi2       =     0.0000
Log likelihood = -3125.4182                     Pseudo R2         =     0.0299

-------------------------------------------------------------------------------------
          emergency |        IRR   Std. Err.      z    P>|z|     [95% Conf. Interval]
--------------------+----------------------------------------------------------------
           strokew1 |
            stroke  |   1.437654   .3707305     1.41   0.159     .8672684    2.383171
              zMoca |   1.068789   .0458264     1.55   0.121     .9826412    1.162489
                    |
           longterm |
 Yes longterm illness  |   1.817805   .1434494     7.57   0.000     1.557313     2.12187
                age |   1.001628   .0053026     0.31   0.759     .9912887    1.012075
                    |
                sex |
            Female  |   .9602327   .0728892    -0.53   0.593     .8274916    1.114267
                    |
               edu3 |
     secondary edu  |   .9717456   .0939108    -0.30   0.767     .8040652    1.174394
   third level edu  |   1.122789   .1220436     1.07   0.287     .9073509     1.38938
                    |
               emp2 |
          Employed  |   1.013597   .0973149     0.14   0.888     .8397342    1.223457
                    |
            medcard |
medcard/gp visit card  |   1.357385    .125377     3.31   0.001      1.13261    1.626767
                    |
        Disability2 |
        Disability  |   1.559045   .1682998     4.11   0.000     1.261743    1.926399
        MHcesd_capi |   1.017002   .0050314     3.41   0.001     1.007189    1.026912
              _cons |   .1036375   .0380454    -6.18   0.000     .0504708    .2128106
--------------------+----------------------------------------------------------------
           /lnalpha |   1.072501   .0801795                      .9153518     1.22965
--------------------+----------------------------------------------------------------
              alpha |    2.92268   .2343391                      2.497654    3.420032
-------------------------------------------------------------------------------------
LR test of alpha=0: chibar2(01) = 610.75              Prob >= chibar2 = 0.000
```

3a.Nights in hospital - original model

```
. nbreg nights i.strokew1 zMoca age i.sex i.edu3 i.emp2 i.medcard i.Disability2 MHcesd_capi, irr
nolog

Negative binomial regression                    Number of obs     =      5,758
                                                LR chi2(10)       =     100.85
Dispersion     = mean                           Prob > chi2       =     0.0000
Log likelihood = -4043.7539                     Pseudo R2         =     0.0123

------------------------------------------------------------------------------------
              nights |        IRR   Std. Err.      z    P>|z|     [95% Conf. Interval]
---------------------+--------------------------------------------------------------
            strokew1 |
             stroke  |   1.930541   .8994416     1.41   0.158     .7746455    4.811217
               zMoca |   1.047743   .0662378     0.74   0.461       .92564    1.185952
                 age |   1.021044   .0088188     2.41   0.016     1.003905    1.038475
                     |
                 sex |
             Female  |   .9332921   .1110405    -0.58   0.562     .7391694    1.178396
                     |
                edu3 |
       secondary edu |   1.233323   .1891828     1.37   0.172     .9130801    1.665885
     third level edu |   1.251192   .2208183     1.27   0.204     .8853166    1.768274
                     |
                emp2 |
           Employed  |   .7813591   .1203808    -1.60   0.109     .5777096    1.056798
                     |
             medcard |
medcard/gp visit card  |   1.518647   .2199657     2.88   0.004     1.143315    2.017193
                     |
         Disability2 |
         Disability  |   2.106449   .3963532     3.96   0.000     1.456758    3.045892
         MHcesd_capi |     1.0189   .0088877     2.15   0.032     1.001628    1.036469
               _cons |   .0973825   .0587259    -3.86   0.000     .0298655    .3175357
---------------------+--------------------------------------------------------------
            /lnalpha |   2.852873   .0487549                      2.757315    2.948431
---------------------+--------------------------------------------------------------
               alpha |   17.33752   .8452886                      15.75748    19.07599
------------------------------------------------------------------------------------
LR test of alpha=0: chibar2(01) = 1.0e+04             Prob >= chibar2 = 0.000
```

3b.Nights in hospital - model including longterm illness

```
. nbreg nights i.strokew1 zMoca i.longterm age i.sex i.edu3 i.emp2 i.medcard i.Disability2
MHcesd_capi, irr nolog

Negative binomial regression                    Number of obs     =      5,753
                                                LR chi2(11)       =     136.55
Dispersion     = mean                           Prob > chi2       =     0.0000
Log likelihood = -4019.3393                     Pseudo R2         =     0.0167

--------------------------------------------------------------------------------------
              nights |        IRR   Std. Err.      z    P>|z|     [95% Conf. Interval]
---------------------+----------------------------------------------------------------
            strokew1 |
             stroke  |   1.792692   .8231025     1.27   0.204     .7289243    4.408889
               zMoca |   1.064631   .0674806     0.99   0.323     .9402574    1.205457
                     |
            longterm |
 Yes longterm illness  |   2.078446   .2570556     5.92   0.000     1.631041    2.648577
                 age |   1.025841   .0088873     2.94   0.003      1.00857    1.043409
                     |
                 sex |
             Female  |   .9534505    .112887    -0.40   0.687     .7559922    1.202483
                     |
                edu3 |
       secondary edu  |   1.204587    .183036     1.22   0.221     .8943318    1.622473
     third level edu  |   1.156953   .2031283     0.83   0.406     .8201041     1.63216
                     |
                emp2 |
           Employed  |   .8838343   .1365227    -0.80   0.424     .6529634    1.196335
                     |
             medcard |
medcard/gp visit card  |     1.4375   .2066904     2.52   0.012     1.084472     1.90545
                     |
         Disability2 |
          Disability  |   1.680238   .3195497     2.73   0.006     1.157413    2.439233
         MHcesd_capi |   1.012504   .0086243     1.46   0.145      .995741    1.029549
               _cons |    .054652   .0333283    -4.77   0.000     .0165394    .1805891
---------------------+----------------------------------------------------------------
            /lnalpha |    2.81531   .0491421                      2.718993    2.911627
---------------------+----------------------------------------------------------------
               alpha |   16.69835   .8205916                      15.16504    18.38668
--------------------------------------------------------------------------------------
LR test of alpha=0: chibar2(01) = 9877.93              Prob >= chibar2 = 0.000
```

4a.Outpatient visits - original model

```
. nbreg outpatient i.strokew1 zMoca age i.sex i.edu3 i.emp2 i.medcard i.Disability2 MHcesd_capi,
irr nolog

Negative binomial regression                    Number of obs     =      5,758
                                                LR chi2(10)       =     320.19
Dispersion     = mean                           Prob > chi2       =     0.0000
Log likelihood = -8323.6887                     Pseudo R2         =     0.0189

------------------------------------------------------------------------------------
           outpatient |        IRR   Std. Err.      z    P>|z|     [95% Conf. Interval]
----------------------+-------------------------------------------------------------
             strokew1 |
               stroke  |   1.491691   .2666236     2.24   0.025     1.050838    2.117493
                zMoca |   .9233992   .0251754    -2.92   0.003     .8753515    .9740841
                  age |   .9991229   .0033752    -0.26   0.795     .9925296     1.00576
                      |
                  sex |
               Female  |    .970144   .0461093    -0.64   0.524      .883853     1.06486
                      |
                 edu3 |
        secondary edu  |   .9581131   .0588409    -0.70   0.486     .8494575    1.080667
      third level edu  |   1.048307    .072027     0.69   0.492     .9162296    1.199424
                      |
                 emp2 |
             Employed  |   .6883726   .0408307    -6.30   0.000     .6128225    .7732367
                      |
              medcard |
medcard/gp visit card  |   1.308491   .0735854     4.78   0.000      1.17193    1.460964
                      |
          Disability2 |
          Disability  |   1.786977     .12974     8.00   0.000     1.549955    2.060244
          MHcesd_capi |   1.021031   .0035114     6.05   0.000     1.014172    1.027936
                _cons |   1.027715   .2380878     0.12   0.906     .6526458    1.618334
----------------------+-------------------------------------------------------------
             /lnalpha |   .7918733   .0349612                      .7233506     .860396
----------------------+-------------------------------------------------------------
                alpha |   2.207528   .0771779                      2.061328    2.364097
------------------------------------------------------------------------------------
LR test of alpha=0: chibar2(01) = 5125.52            Prob >= chibar2 = 0.000
```

4b.Outpatient visits - model including longterm illness

```
. nbreg outpatient i.strokew1 zMoca i.longterm age i.sex i.edu3 i.emp2 i.medcard i.Disability2
MHcesd_capi, irr nolog

Negative binomial regression                    Number of obs     =      5,753
                                                LR chi2(11)       =     643.70
Dispersion     = mean                           Prob > chi2       =     0.0000
Log likelihood = -8151.6442                     Pseudo R2         =     0.0380

------------------------------------------------------------------------------------
           outpatient |        IRR   Std. Err.      z    P>|z|     [95% Conf. Interval]
----------------------+-------------------------------------------------------------
             strokew1 |
              stroke  |   1.410321   .2403202     2.02   0.044     1.009886    1.969534
                zMoca |   .9332813   .0245787    -2.62   0.009     .8863301    .9827196
                      |
             longterm |
 Yes longterm illness |   2.401465   .1150536    18.29   0.000     2.186228    2.637893
                  age |    1.00328   .0032902     1.00   0.318      .996852    1.009749
                      |
                  sex |
              Female  |   .9628969   .0444146    -0.82   0.412      .879665    1.054004
                      |
                 edu3 |
       secondary edu  |   .9677825   .0574478    -0.55   0.581       .86149     1.08719
     third level edu  |   1.032862   .0685443     0.49   0.626     .9068876    1.176335
                      |
                 emp2 |
            Employed  |   .7984812   .0466383    -3.85   0.000     .7121101    .8953282
                      |
              medcard |
medcard/gp visit card |   1.216473   .0668381     3.57   0.000      1.09228    1.354788
                      |
          Disability2 |
          Disability  |    1.41033   .0999349     4.85   0.000     1.227454    1.620452
          MHcesd_capi |   1.013218    .003349     3.97   0.000     1.006675    1.019803
                _cons |   .5439719   .1236483    -2.68   0.007     .3484119    .8492979
----------------------+-------------------------------------------------------------
             /lnalpha |   .6490419   .0367526                       .5770082    .7210756
----------------------+-------------------------------------------------------------
                alpha |   1.913706   .0703336                       1.780703    2.056644
------------------------------------------------------------------------------------
LR test of alpha=0: chibar2(01) = 4343.80             Prob >= chibar2 = 0.000
```

5a.Rehab services used - 3 services (new variable: OT, Psychology and Physiotherapy)

```
. logit SSused3 i.strokew1 zMoca age i.sex i.edu3 i.emp2 i.medcard i.Disability2 MHcesd_capi, or
nolog

Logistic regression                             Number of obs     =      5,760
                                                LR chi2(10)       =     260.77
                                                Prob > chi2       =     0.0000
Log likelihood = -1259.2078                     Pseudo R2         =     0.0938

------------------------------------------------------------------------------------
              SSused3 | Odds Ratio   Std. Err.      z    P>|z|     [95% Conf. Interval]
----------------------+-------------------------------------------------------------
             strokew1 |
               stroke  |   1.247889   .4231788     0.65   0.514      .641978     2.42567
                zMoca |   .9854331   .0581176    -0.25   0.804     .8778617    1.106186
                  age |   .9876777   .0072565    -1.69   0.091     .9735571    1.002003
                      |
                  sex |
               Female  |   1.139399    .129593     1.15   0.251     .9117204    1.423934
                      |
                 edu3 |
        secondary edu  |   1.447394   .2076175     2.58   0.010     1.092668    1.917278
      third level edu  |   1.763759   .2863017     3.50   0.000     1.283122    2.424433
                      |
                 emp2 |
             Employed  |   .6002524   .0949352    -3.23   0.001     .4402596    .8183875
                      |
              medcard |
medcard/gp visit card  |   2.697211   .3800268     7.04   0.000     2.046369    3.555053
                      |
          Disability2 |
           Disability  |   2.593357   .3499073     7.06   0.000     1.990739    3.378394
          MHcesd_capi |   1.039322   .0066217     6.05   0.000     1.026425    1.052382
                _cons |   .0424572   .0217686    -6.16   0.000     .0155426    .1159789
------------------------------------------------------------------------------------
```

5b. Rehab services used (3 services) - model including longterm illness

```
. logit SSused3 i.strokew1 zMoca i.longterm age i.sex i.edu3 i.emp2 i.medcard i.Disability2
MHcesd_capi, or nolog

Logistic regression                              Number of obs     =      5,755
                                                 LR chi2(11)       =     313.11
                                                 Prob > chi2       =     0.0000
Log likelihood = -1232.7022                      Pseudo R2         =     0.1127

------------------------------------------------------------------------------------------
               SSused3 | Odds Ratio   Std. Err.      z    P>|z|     [95% Conf. Interval]
-----------------------+------------------------------------------------------------------
              strokew1 |
               stroke  |    1.15439   .3893762     0.43   0.670     .5959938    2.235955
                 zMoca |   .9964729    .059103    -0.06   0.952     .8871128    1.119315
                       |
              longterm |
 Yes longterm illness  |   2.414493   .2995352     7.11   0.000     1.893339    3.079099
                   age |   .9898175   .0073056    -1.39   0.166     .9756019     1.00424
                       |
                   sex |
               Female  |    1.13947   .1301702     1.14   0.253     .9108858    1.425418
                       |
                  edu3 |
        secondary edu  |   1.459999   .2107668     2.62   0.009     1.100202    1.937461
      third level edu  |   1.748068   .2855369     3.42   0.001     1.269169    2.407672
                       |
                  emp2 |
             Employed  |   .6657387   .1061474    -2.55   0.011     .4870636    .9099591
                       |
               medcard |
medcard/gp visit card  |   2.516501   .3551644     6.54   0.000     1.908373    3.318415
                       |
           Disability2 |
           Disability  |   1.965227   .2731058     4.86   0.000     1.496656    2.580497
           MHcesd_capi |   1.033779   .0066755     5.14   0.000     1.020778    1.046946
                 _cons |   .0256163   .0133742    -7.02   0.000     .0092068    .0712731
------------------------------------------------------------------------------------------
```

6a.Rehab services used - 2 services only (original variable: OT and Psychology)

```
. logit RehabUsed2 i.strokew1 zMoca age i.sex i.edu3 i.emp2 i.medcard i.Disability2 MHcesd_capi,
or nolog

Logistic regression                               Number of obs     =      5,760
                                                  LR chi2(10)       =     160.85
                                                  Prob > chi2       =     0.0000
Log likelihood = -533.39536                       Pseudo R2         =     0.1310

------------------------------------------------------------------------------------------
            RehabUsed2 | Odds Ratio   Std. Err.      z    P>|z|     [95% Conf. Interval]
-----------------------+------------------------------------------------------------------
              strokew1 |
               stroke  |   3.023982   1.210921     2.76   0.006      1.37951    6.628779
                 zMoca |   .9380939   .0918678    -0.65   0.514     .7742619    1.136592
                   age |   .9660501   .0117159    -2.85   0.004     .9433582    .9892879
                       |
                   sex |
               Female  |     .98947   .1873641    -0.06   0.955     .6826857    1.434117
                       |
                  edu3 |
        secondary edu  |   1.573118   .3827416     1.86   0.063     .9764793     2.53431
      third level edu  |   2.043387   .5582288     2.62   0.009     1.196224    3.490509
                       |
                  emp2 |
             Employed  |   .4544699   .1282731    -2.79   0.005     .2613698    .7902326
                       |
               medcard |
medcard/gp visit card  |   2.865971   .6841755     4.41   0.000     1.795022    4.575872
                       |
           Disability2 |
           Disability  |   3.386041   .7127287     5.79   0.000     2.241416    5.115191
           MHcesd_capi |   1.048094   .0098247     5.01   0.000     1.029013    1.067528
                 _cons |   .0442429   .0373463    -3.69   0.000     .0084591    .2313985
------------------------------------------------------------------------------------------
```

6b.Rehab services used (2 services) - model including longterm illness

```
. logit RehabUsed2 i.strokew1 zMoca i.longterm age i.sex i.edu3 i.emp2 i.medcard i.Disability2
MHcesd_capi, or nolog

Logistic regression                              Number of obs     =      5,755
                                                 LR chi2(11)       =     185.15
                                                 Prob > chi2       =     0.0000
Log likelihood = -521.13046                      Pseudo R2         =     0.1508

------------------------------------------------------------------------------------------
            RehabUsed2 | Odds Ratio   Std. Err.      z    P>|z|     [95% Conf. Interval]
-----------------------+------------------------------------------------------------------
              strokew1 |
               stroke  |   2.809574   1.118978     2.59   0.009     1.287151    6.132693
                 zMoca |   .9468643   .0929152    -0.56   0.578     .7811957    1.147666
                       |
              longterm |
 Yes longterm illness  |   2.950673    .678337     4.71   0.000      1.88034    4.630264
                   age |    .968969   .0117659    -2.60   0.009     .9461805    .9923063
                       |
                   sex |
               Female  |   .9991877   .1894041    -0.00   0.997     .6891202    1.448769
                       |
                  edu3 |
        secondary edu  |   1.583393   .3870808     1.88   0.060     .9806219    2.556677
      third level edu  |   2.005828   .5500773     2.54   0.011      1.17182    3.433419
                       |
                  emp2 |
             Employed  |   .5127925   .1452453    -2.36   0.018     .2943365    .8933862
                       |
               medcard |
medcard/gp visit card  |    2.61402   .6224874     4.04   0.000     1.639112     4.16878
                       |
           Disability2 |
           Disability  |   2.444502   .5277837     4.14   0.000     1.601071    3.732246
           MHcesd_capi |   1.042332    .009845     4.39   0.000     1.023214    1.061808
                 _cons |   .0223162   .0192626    -4.41   0.000     .0041105    .1211569
------------------------------------------------------------------------------------------
```
