# Supplementary material for: The impact of stroke, cognitive function and post-stroke cognitive impairment (PSCI) on healthcare utilisation in Ireland: a cross-sectional nationally representative study
Source: BMC Health Serv Res. 2022 Mar 29;22:414. doi: 10.1186/s12913-022-07837-2 (PMC8962254; doi:10.1186/s12913-022-07837-2)
Supplement: Supplementary file 2 — Additional file 2: Table 1. Demographic variables all comparisons. [file 12913_2022_7837_MOESM2_ESM.docx]

**File name:** Additional File 2 Table 1 Demographic variables_all comparisons

**File format:** Microsoft Word (.docx)

**Title of data:** Supplementary Table 1 Associations between demographic and health variables, and stroke and cognitive status

**Description of data:** Additional File 2 presents the results of the unadjusted associations between stroke/cognitive status groups and the demographic and health-related variables. Statistically significant differences between groups are indicated.

**Supplementary Table 1 Associations between demographic and health variables, and stroke and cognitive status**

|  | No stroke | | Stroke | |  | |
| --- | --- | --- | --- | --- | --- | --- |
|  | **NCI**  **(n= 4020; 68.6%)** | **CI**  **(n= 1747; 29.8%)** | **NCI**  **(n= 42; 0.7%)** | **CI**  **(n= 50; 0.9%)** | **Test statistic** | |
|  | **Mean (SD)** | **Mean (SD)** | **Mean (SD)** | **Mean (SD)** | **F** | **P value** |
| Montreal Cognitive Assessment Score (MoCA) (n=5859) | 26.7 (1.81)^*$#+^  n= 4020 (68.6%) | 20.2 (3.10)^*^**^$^**^#+^  n= 1747 (29.8%) | 25.9 (1.61)^*$^**^#^**^+^  n= 42 (0.7%) | 18.6 (4.53)^*$#^**^+^**  n= 50 (0.9%) | 3303.95 | <0.001 |
| Age* (n= 5849) | 61.3 (8.09)**^*^**^$#+^  n= 4015 (68.7%) | 66.3 (9.38)^*^**^$^**^+^  n= 1742 (29.8%) | 67.3 (7.05)^*^**^#^**^+^  n= 42 (0.7%) | 71.0 (8.93)^*$#^**^+^**  n= 50 (0.8%) | 158.51 | <0.001 |
|  | **n (%)** | **n (%)** | **n (%)** | **n (%)** | **Cramer’s V** | **P value** |
| Sex  Male  Female | 1832 (45.6)  2188 (54.4) | 807 (46.2)  940 (53.8) | 22 (52.4)  20 (47.6) | 28 (56.0)  22 (44.0) | 0.023 | 0.392 |
| Education  None/primary school  Secondary/high school  Third level/university | 674 (16.8)**^*^**^$+^  1696 (42.2)  1650 (41.0) | 812 (46.5)^*^**^$^**^#^  678 (38.9)  255 (14.6) | 12 (28.5)^$^**^#^**^+^  17 (40.5)  13 (31.0) | 25 (50.0)^*#^**^+^**  20 (40.0)  5 (10.0) | 0.244 | <0.001 |
| Marital status  Not married  Married | 973 (24.2)**^*^**^$+^  3047 (75.8) | 617 (35.3)^*^**^$^**  1130 (64.7) | 11 (26.2)  31 (73.8) | 22 (44.0)^*^**^+^**  28 (56.0) | 0.118 | <0.001 |
| Living situation  Living alone  Living with others | 700 (17.4)**^*^**^$+^  3320 (82.6) | 446 (25.5)^*^**^$^**  1301 (74.5) | 8 (19.0)  34 (81.0) | 17 (34.0)^*^**^+^**  33 (66.0) | 0.098 | <0.001 |
| Geographical location  Urban (town/city)  Rural | 2263 (56.3)**^*^**^$^  1754 (43.7) | 809 (46.4)^*^**^$^**^#^  936 (53.6) | 27 (64.3)^$^**^#^**  15 (35.7) | 28 (56.0)  22 (44.0) | 0.093 | <0.001 |
| Employment status  Unemployed  Employed | 2258 (56.2)**^*^**^$#+^  1762 (43.8) | 1298 (74.3)^*^**^$^**^+^  449 (25.7) | 35 (83.3)^*^**^#^**  7 (16.7) | 45 (90.0)^*$^**^+^**  5 (10.0) | 0.182 | <0.001 |
| Medical card  No  Yes | 2602 (64.8)**^*^**^$#+^  1414 (35.2) | 625 (35.8)^*^**^$^**^+^  1122 (64.2) | 13 (30.9)^*^**^#^**^+^  29 (69.1) | 4 (8.0)^*$#^**^+^**  46 (92.0) | 0.284 | <0.001 |
| Private insurance  No  Yes | 1207 (30.0)**^*^**^$+^  2810 (70.0) | 893 (51.2)^*^**^$^**^#^  853 (48.8) | 14 (33.3)^$^**^#^**^+^  28 (66.7) | 32 (64.0)^*#^**^+^**  18 (36.0) | 0.207 | <0.001 |
| Disability status  No disability  Disability | 3.686 (91.7)**^*^**^$#+^  334 (8.3) | 1440 (82.4)^*^**^$^**^#+^  307 (17.6) | 29 (69.1)^*$^**^#^**  13 (30.9) | 29 (58.0)^*$^**^+^**  21 (42.0) | 0.168 | <0.001 |
| Body Mass Index (BMI)  Normal weight  Overweight/obese | 951 (23.7)**^*^**^$^  3060 (76.3) | 366 (21.1)^*^**^$^**  1372 (78.9) | 10 (23.8)  32 (76.2) | 7 (14.6)  41 (85.4) | 0.034 | 0.081 |
| Smoking status  Non-smoker/ex-smoker  Current smoker | 3426 (85.2)**^*^**^$^  594 (14.8) | 1419 (81.2)^*^**^$^**  328 (18.8) | 37 (88.1)  5 (11.9) | 41 (82.0)  9 (18.0) | 0.051 | 0.002 |
| Physical activity level  Low  Moderate/High | 1077 (27.0)**^*^**^$+^  2908 (73.0) | 630 (36.3)^*^**^$^**^+^  1103 (63.7) | 17 (40.5)  25 (59.5) | 28 (56.0)^*$^**^+^**  22 (44.0) | 0.108 | <0.001 |
| CVD conditions  No CVD conditions  At least one CVD condition | 1520 (37.8)**^*^**^$#+^  2500 (62.2) | 566 (32.4)^*^**^$^**^#^  1181 (67.6) | 2 (4.8)^*$^**^#^**^+^  40 (95.2) | 11 (22.0)^*#^**^+^**  39 (78.0) | 0.080 | <0.001 |
| Medications  No Polypharmacy  Polypharmacy (5+ medications) | 3493 (87.3)**^*^**^$#+^  509 (12.7) | 1262 (73.0)^*^**^$^**^#+^  466 (27.0) | 18 (42.9)^*$^**^#^**  24 (57.1) | 25 (50.0)^*$^**^+^**  25 (50.0) | 0.208 | <0.001 |
| Depression  None/mild  Moderate/severe | 3670 (92.6)**^*^**^$+^  294 (7.4) | 1509 (87.5)^*^**^$^**  215 (12.5) | 33 (84.6)  6 (15.4) | 40 (83.3)^*^**^+^**  8 (16.7) | 0.086 | <0.001 |
| Anxiety  None/mild  Moderate/severe | 2834 (77.2)**^*^**^$^  836 (22.8) | 1033 (71.7)^*^**^$^**  407 (28.3) | 23 (63.9)  13 (36.1) | 22 (64.7)  12 (35.3) | 0.065 | <0.001 |

Results are based on Chi-Square tests (categorical variables) and Analysis of Variance (ANOVA) tests (continuous variables).

*Denotes a statistically significant difference (p≤ 0.05) between this category and the reference category (No stroke/ NCI).

$ Denotes a statistically significant difference (p≤ 0.05) between this category and the No stroke/ CI category.

# Denotes a statistically significant difference (p≤ 0.05) between this category and the Stroke/ NCI category.

+ Denotes a statistically significant difference (p≤ 0.05) between this category and the Stroke/ CI category.

NCI = No Cognitive Impairment; CI = Cognitive Impairment; SD = Standard Deviation; CVD = Cardiovascular Disease
