# Supplementary material for: The impact of stroke, cognitive function and post-stroke cognitive impairment (PSCI) on healthcare utilisation in Ireland: a cross-sectional nationally representative study
Source: BMC Health Serv Res. 2022 Mar 29;22:414. doi: 10.1186/s12913-022-07837-2 (PMC8962254; doi:10.1186/s12913-022-07837-2)
Supplement: Supplementary file 3 — Additional file 3: Table 2. HSU all comparisons. [file 12913_2022_7837_MOESM3_ESM.docx]

**File name:** Additional File 3 Table 2 HSU_all comparisons

**File format:** Microsoft Word (.docx)

**Title of data:** Supplementary Table 2 Associations between healthcare utilisation variables, and stroke and cognitive status

**Description of data:** Additional File 3 presents the results of the unadjusted associations between stroke/cognitive status groups and the healthcare utilisation variables. Statistically significant differences between groups are indicated.

**Supplementary Table 2 Associations between healthcare utilisation variables, and stroke and cognitive function**

|  | No stroke | | Stroke | |  |  |
| --- | --- | --- | --- | --- | --- | --- |
| Healthcare utilisation variables | **NCI**  **(n= 4020; 68.6%)** | **CI**  **(n= 1747; 29.8%)** | **NCI**  **(n= 42; 0.7%)** | **CI**  **(n= 50; 0.9%)** |  |  |
|  | **N used service (%)**  **Mean (SD)**  **Median (range)** | **N used service (%)**  **Mean (SD)**  **Median (range)** | **N used service (%)**  **Mean (SD)**  **Median (range)** | **N used service (%)**  **Mean (SD)**  **Median (range)** | **Kruskal-Wallis test** | **P value** |
| GP visits^a^ (n= 5851) | 3447 (85.7)  3.2 (3.33)**^*^**^$#+^  2 (0-25) | 1592 (91.1)  4.7 (4.65)^*^**^$^**^#+^  4 (0-25) | 39 (92.9)  6.9 (6.77)^*$^**^#^**  4 (0-25) | 48 (96.0)  7.3 (6.07)**^*^**^$^**^+^**  4 (0-25) | 215.382 | <0.001 |
| Emergency visits^b^ (n=5856) | 563 (14.0)  0.20 (0.62)**^*^**^$#+^  0 (0-6) | 299 (17.1)  0.27 (0.74)^*^**^$^**^#^  0 (0-6) | 12 (28.6)  0.62 (1.32)^*$^**^#^**  0 (0-6) | 12 (24.0)  0.42 (0.88)^*^**^+^**  0 (0-4) | 8.184 | 0.042 |
| Number of nights in hospital^c^ (n=5857) | 449 (11.2)  0.54 (1.90)**^*^**^$+^  0 (0-10) | 259 (14.8)  0.84 (2.39)^*^**^$^**  0 (0-10) | 8 (19.0)  1.52 (3.42)  0 (0-10) | 15 (30.0)  2.34 (3.94)^*^**^+^**  0 (0-10) | 11.745 | 0.008 |
| Outpatient visits^c^ (n=5857) | 1692 (42.1)  1.17 (2.09)**^*^**^$#^  0 (0-10) | 769 (44.0)  1.32 (2.32)^*^**^$^**^#^  0 (0-10) | 26 (61.9)  3.10 (3.57)^*$^**^#^**  2 (0-10) | 23 (46.0)  1.88 (2.90)  0 (0-10) | 15.224 | 0.002 |
|  | | | | | | |
|  | **N (%)** | **N (%)** | **N (%)** | **N (%)** | **Cramer’s V** | **P value** |
| Physiotherapy (PT)  Not used  Used service | 3834 (95.4)**^*^**^$^  186 (4.6) | 1636 (93.7)^*^**^$^**  111 (6.3) | 40 (95.2)  2 (4.8) | 45 (90.0)  5 (10.0) | 0.041 | 0.015^d^ |
| Occupational therapy (OT)  Not used  Used service | 3977 (98.9)**^*^**^#+^  43 (1.1) | 1721 (98.5)**^$^**^#+^  26 (1.5) | 39 (92.9)^*$^**^#^**  3 (7.1) | 44 (88.0)^*$^**^+^**  6 (12.0) | 0.098 | <0.001 |
| Psychology (PSY)  Not used  Used service | 3980 (99.0)  40 (1.0) | 1730 (99.0)  17 (1.0) | 41 (97.6)  1 (2.4) | 50 (100.0)  0 (0.0) | 0.015 | 0.574^d^ |
| At least 1 rehabilitation service used (OT/ PSY)  Not used  Used services | 3782 (94.1)**^*^**^$+^  238 (5.9) | 1608 (92.0)^*^**^$^**^+^  139 (8.0) | 38 (90.5)  4 (9.5) | 41 (82.0)^*$^**^+^**  9 (18.0) | 0.057 | <0.001^d^ |

Results are based on Chi-Square tests (categorical variables) and the Kruskal-Wallis test for non-normally distributed outcomes (continuous variables).

^*^Denotes a statistically significant difference (p≤ 0.05) between this category and the reference category (No stroke/ NCI).

^$^Denotes a statistically significant difference (p≤ 0.05) between this category and the No stroke/ CI category.

^#^Denotes a statistically significant difference (p≤ 0.05) between this category and the Stroke/ NCI category.

^+^Denotes a statistically significant difference (p≤ 0.05) between this category and the Stroke/ CI category.

NCI = No Cognitive Impairment; CI = Cognitive Impairment; SD = Standard Deviation; GP = General Practitioner

^a^ variable truncated at 25 visits in the public TILDA dataset; 5126 participants had at least one visit to the GP.

^b^ variable truncated at 6 visits in the public TILDA dataset; 886 participants had at least one visit to emergency services.

^c^ variable truncated at 10 visits in the public TILDA dataset; 731 participants spent at least one night in hospital; 2510 participants had at least one visit to outpatient services.

^d^ Fisher’s exact text (adjusted for small samples)
