# Supplementary material for: The impact of stroke, cognitive function and post-stroke cognitive impairment (PSCI) on healthcare utilisation in Ireland: a cross-sectional nationally representative study
Source: BMC Health Serv Res. 2022 Mar 29;22:414. doi: 10.1186/s12913-022-07837-2 (PMC8962254; doi:10.1186/s12913-022-07837-2)
Supplement: Supplementary file 4 — Additional file 4: Table 3. Unadjusted and adjusted associations HSU. [file 12913_2022_7837_MOESM4_ESM.docx]

**File name:** Additional File 4 Table 3 Unadjusted and adjusted associations HSU

**File format:** Microsoft Word (.docx)

**Title of data:** Supplementary Table 3 Unadjusted and adjusted associations between healthcare utilisation, stroke and cognitive impairment

**Description of data:** Additional File 4 presents the results of the unadjusted and adjusted regressions, using the dichotomised Montreal Cognitive Assessment (MoCA) variable (scores <24 indicate cognitive impairment).

**Supplementary Table 3 Unadjusted and adjusted associations between healthcare utilisation, stroke and cognitive impairment**

| Healthcare type | Exposure | Unadjusted model  IRR (95% CI) | P value | Exposure | Fully adjusted model  IRR (95% CI) | P value |
| --- | --- | --- | --- | --- | --- | --- |
| GP visits^a^ | | | | | | |
|  | **Stroke** (n=8164) | 2.04 (1.75-2.38) | <0.001 | **Stroke** | 1.28 (1.08-1.52) | 0.004 |
|  | **Cognitive impairment** (n=5851) | 1.50 (1.42-1.57) | <0.001 | **Cognitive impairment** | 1.13 (1.08-1.19) | <0.001 |
|  | **Stroke*cognitive impairment** (n=5851) | 0.71 (0.49-1.01) | 0.059 | **Stroke*cognitive impairment** | 0.86 (0.61-1.20) | 0.361 |
| Emergency visits^b^ | | | | | | |
|  | **Stroke** (n= 8167) | 2.93 (1.96-4.39) | <0.001 | **Stroke** | 1.59 (0.95-2.65) | 0.076 |
|  | **Cognitive impairment** (n=5856) | 1.35 (1.16-1.58) | <0.001 | **Cognitive impairment** | 1.11 (0.94-1.32) | 0.230 |
|  | **Stroke*cognitive impairment** (n=5856) | 0.50 (0.19-1.32) | 0.163 | **Stroke*cognitive impairment** | 0.69 (0.25-1.90) | 0.476 |
| Nights in hospital^c^ | | | | | | |
|  | **Stroke** (n=8172) | 3.88 (1.83-8.23) | <0.001 | **Stroke** | 1.92 (0.77-4.79) | 0.160 |
|  | **Cognitive impairment** (n=5857) | 1.58 (1.23-2.03) | <0.001 | **Cognitive impairment** | 1.17 (0.90-1.53) | 0.244 |
|  | **Stroke*cognitive impairment** (n=5857) | 1.00 (0.62-6.18) | 1.000 | **Stroke*cognitive impairment** | 1.37 (0.22-8.41) | 0.736 |
| Outpatient visits^d^ | | | | | | |
|  | **Stroke** (n=8168) | 2.05 (1.51-2.79) | <0.001 | **Stroke** | 1.48 (1.04-2.10) | 0.028 |
|  | **Cognitive impairment** (n=5857) | 1.13 (1.02-1.25) | 0.020 | **Cognitive impairment** | 0.92 (0.83-1.03) | 0.154 |
|  | **Stroke*cognitive impairment** (n=5857) | 0.54 (0.26-1.09) | 0.085 | **Stroke*cognitive impairment** | 0.63 (0.31-1.27) | 0.195 |
| Healthcare type | **Exposure** | **Unadjusted model**  **OR (95% CI)** | **P value** | **Exposure** | **Fully adjusted model**  **OR (95% CI)** | **P value** |
| Rehabilitation services used^e^ | | | | | | |
|  | **Stroke** (n= 8175) | 2.70 (1.68-4.34) | <0.001 | **Stroke** | 1.24 (0.64-2.41) | 0.521 |
|  | **Cognitive impairment** (n= 5859) | 1.42 (1.15-1.75) | 0.001 | **Cognitive impairment** | 0.99 (0.77-1.27) | 0.928 |
|  | **Stroke*cognitive impairment** (n=5859) | 1.52 (0.42-5.44) | 0.521 | **Stroke*cognitive impairment** | 2.73 (0.62-12.00) | 0.183 |

Full model adjusted for health and demographic factors (stroke status, cognitive function status, age, sex, education, employment, medical card, disability and depression).

Cognitive impairment is based on the dichotomised Montreal Cognitive Assessment (MoCA) variable (scores <24 indicate cognitive impairment).

Stroke*cognitive impairment tests whether there is an interaction between stroke status and cognitive function (impaired versus unimpaired).

IRR = Incidence-rate ratio; OR = Odds Ratio; GP = General Practitioner

**^a^** Fully adjusted model (n=5753)

**^b^** Fully adjusted model (n=5757)

**^c d^** Fully adjusted model (n=5758)

**^e^** Fully adjusted model (n=5760)
